# Neuroscientific Analysis of Logo Design: Implications for Luxury Brand Marketing

**DOI:** 10.3390/bs15040502

**Published:** 2025-04-09

**Authors:** Hedda Martina Šola, Sarwar Khawaja, Fayyaz Hussain Qureshi

**Affiliations:** 1 Oxford Business College (SK Research), Macclesfield House, New Road, Oxford OX1 1BY, UK; advice@obc.ac.uk (S.K.); fayyaz.qureshi@obc.ac.uk (F.H.Q.); 2 Institute for Neuromarketing & Intellectual Property, Jurja Ves III spur no 4, 10000 Zagreb, Croatia

**Keywords:** neuromarketing, AI EEG, AI eye tracking, implicit testing, neurobranding, memory, neuroscience of logos, luxury brand marketing, cognitive engagement, visual attention

## Abstract

This study examines the influence of dynamic and verbal elements in logo design on consumer behaviour in the luxury retail sector using advanced neuroscience technology (Predict v.1.0) and traditional cognitive survey methods. AI-powered eye tracking (*n* = 255,000), EEG technology (*n* = 45,000), implicit testing (*n* = 9000), and memory testing (*n* = 7000) were used to predict human behaviour. Qualitative cognitive surveys (*n* = 297), saliency map analysis, and emotional response evaluation were employed to analyse three distinct logo designs. The results indicate that logos with prominent dynamic elements, particularly visually distinct icons, demonstrate superior performance in capturing and maintaining viewer attention compared with static designs. A strong correlation was found between cognitive demand and engagement, suggesting that dynamic elements enhance emotional connections and brand recall. However, the effectiveness of dynamic features varied, with more pronounced elements yielding better results for industry associations and premium market alignment. This study, combining advanced neuroscience technology with traditional cognitive survey methods, makes significant contributions to the field and opens up new avenues for research and application. The findings provide valuable insights for luxury brand managers in optimising logo designs to enhance emotional connection and brand perception and improve academia by providing powerful tools for understanding and predicting human responses to visual stimuli.

## 1. Introduction

Brand design elements, such as logo shape, brand name, typography, and colour, significantly influence consumer perception of a brand, shape their preferences, and contribute to brand equity (Lieven et al., 2015). Dynamic logos and brand communication elements are crucial for enhancing consumer engagement and increasing brand awareness. Typography is integral to this process, as it ensures visual consistency in dynamic logos by maintaining structural axes while accommodating flexibility, thus striking a balance between innovation and brand identity (Lelis et al., 2022). Dynamic colouring, adjusted based on different contexts or consumer interactions, enhances a logo’s appeal. This concept parallels dynamic colouring in graphs, where adaptability plays a vital role in sustaining engagement and capturing attention (Kashyop et al., 2023). Asymmetrical logos are more visually stimulating, enhance perceptions of excitement, and positively influence consumer evaluations and the financial valuations of brands with dynamic, exciting personalities (Luffarelli et al., 2019b). Furthermore, logos with more descriptive elements have a positive impact on brand evaluations, enhance purchase intentions, and improve brand performance. This effect is attributed to their ease of cognitive processing and the stronger sense of authenticity they convey (Luffarelli et al., 2019a). Anthropomorphic logos, which imbue brands with human-like characteristics, can enhance the perceived functional performance of products. In the cosmetic industry, these logos convey efficacy and reliability, resonating with consumers who prioritise effective skincare solutions (Daryanto et al., 2022).

This research study focuses on dynamic logos, which demonstrate greater efficacy than static ones in capturing attention, resulting in increased viewer retention and enhanced brand recall. Through the incorporation of movement or change elements, these logos elicit curiosity and facilitate deeper consumer engagement with the brand (Veloutsou, 2023). These findings underscore the significance of effective logo design in capturing consumer attention and influencing purchasing decisions (Goudis & Skuras, 2021). Eye-tracking and facial recognition technologies are being increasingly utilised to evaluate consumer reactions to logo designs, providing companies with valuable insights to refine their branding strategies and enhance consumer engagement (Neomániová et al., 2019). Eye-tracking studies reveal that attention trajectories strongly predict brand choice, with dynamic logos playing a crucial role in capturing consumer interest during decision-making (Martinovici et al., 2023). Chosen brands experience a “double attention lift”, wherein focus intensifies as consumers approach their final choice, emphasising the importance of visually appealing and memorable logos. By gathering valuable consumer data, brands can refine their marketing strategies and foster more profound and more personalised connections with their audience (Oc et al., 2023). Eye-tracking studies reveal that dynamic elements in branding capture consumer attention, increasing the fixation duration and overall engagement. Dynamic logos capitalise on the dynamic attention theory, which posits that moving stimuli naturally attract more attention than static designs, enhancing brand visibility and consumer interaction (Wooley et al., 2022). Furthermore, these studies provide valuable insights into consumer interactions with dynamic logos by analysing attention spans and fixation points, offering data to guide design enhancements. Advanced algorithms further refine this process by classifying eye movements with precision, enabling brands to assess the real-time effectiveness of dynamic logos and to optimise their impact (Dar et al., 2021). Thus, the following research question (RQ) is explored:

***RQ:***  *Do dynamic elements in logos increase consumer engagement, emotions, and brand industry awareness?*

The objectives of this research study are to investigate the impact of dynamic and verbal elements in logo design on consumer behaviour within the luxury retail sector. Specifically, the study aims to evaluate the impact of various logo designs on consumer attention, engagement, emotional response, and brand recall. The research employs advanced neuroscience technology, including AI-powered eye tracking, EEG, and implicit testing, in conjunction with traditional cognitive survey methods to analyse three distinct logo designs. The study aims to investigate how dynamic elements in logos influence visual attention, cognitive demand, emotional connections, and industry associations, particularly in the context of luxury brand marketing.

### Extending Predictive Neuromarketing Frameworks

The significance of this study lies in its innovative approach to logo evaluation, which integrates cutting-edge neuroscientific methods with traditional marketing research techniques. This multi-method approach offers a more comprehensive understanding of consumer responses to visual branding elements.

This research makes multifaceted contributions to both theory and practice in neuromarketing and luxury branding. It advances prior work by demonstrating how dynamic logo elements elicit measurable variations in attention, engagement, and memory through the integration of AI-based neurotechnologies. Building on empirical frameworks outlined by Karmarkar et al. (2021), who showed that category-congruent stimuli influence visual attention and decision-making, this study illustrates how design congruency in logos similarly reinforces cognitive alignment and brand preference. Furthermore, by using AI-modelled EEG and eye-tracking data from a sample size of over 300,000, the methodology addresses scalability concerns in neuromarketing raised by Bazzani et al. (2020), showing how robust, predictive frameworks can replace cost- and time-intensive live testing without sacrificing neurophysiological validity.

From a cognitive perspective, the study also extends the findings from Trabulsi et al. (2021) on visual saliency, supporting the idea that well-calibrated dynamic features outperform overly complex or ambiguous forms in guiding the attention given to meaningful brand elements. The integration of dynamic iconography in logo design is shown to elevate consumer engagement and brand memorability—a finding aligned with insights by Wong and Park (2023), who emphasised the relevance of dynamic brand visuals in enhancing luxury positioning. Likewise, the strong correlation observed between cognitive demand and emotional engagebment in this study reinforces observations by Vitolo et al. (2021) on the neurophysiological implications of complexity in visual marketing.

Finally, by aligning neurometric predictions with qualitative survey data, the study meets the interdisciplinary standards of consumer neuroscience advocated by Silberstein et al. (2020), who emphasised the necessity of linking subconscious markers with explicit y (consumer interpretation to enhance real-world branding outcomes. This hybrid methodology provides critical validation of the recent findings by Georgiadis et al. (2023), who underscore the importance of combining EEG, eye tracking, and survey responses to capture holistic consumer reactions in the luxury sector.

Unlike prior research studies, which focused largely on advertising campaigns or product-level stimuli, this study addresses a gap by applying neuromarketing principles to the pre-launch evaluation of logo design—a foundational yet underexplored aspect of brand identity. By demonstrating how specific dynamic elements influence memory encoding, attention allocation, and emotional association, this work contributes to theory-building in neurodesign and expands the methodological practices in predictive consumer science. As such, it sets a benchmark for future interdisciplinary research by bridging AI, neuroscience, and visual brand strategy.

Research indicates that dynamic elements in logos can enhance consumer engagement, emotions, and brand awareness. Both self-reported data and eye-tracking measurements demonstrate that consumer engagement significantly influences attitudes towards the brand (Cian et al., 2014). Effective logo design should consider both the dynamism of the logo and the type of product when trying to influence consumer perceptions of quality and to improve market performance positively (Wang et al., 2023). Moreover, emotional arousal towards a brand logo is positively associated with its aesthetic appeal and recall but negatively correlated with symbolic knowledge. Increased familiarity with a brand logo results in quicker response times, improved recall, stronger aesthetic appeal, and more positive emotional reactions, while subjective perceptions of logo complexity influence emotional responses in contrast to objective complexity measures (De Marchis et al., 2018).

## 2. Background and Hypotheses

### 2.1. Literature Review

#### 2.1.1. The Impact of Artificial Intelligence on Logo Analysis

AI technologies enable the analysis of consumer responses to dynamic logos, offering valuable insights for optimising advertising strategies and for enhancing brand engagement (Ahmed et al., 2022). The incorporation of artificial intelligence (AI) in logo analysis significantly transforms the design and development of brand identities by augmenting creativity, efficiency, and accessibility. AI tools streamline the logo creation process, allowing designers to generate diverse options rapidly and cost-effectively while also providing insights into consumer preferences (Adeleye, 2024; Mohamad Roslan & Saad, 2024; Tuwio, 2024). This evolution necessitates a shift in traditional design methodologies, as AI becomes a collaborative partner in the creative process. The integration of AI in design requires careful consideration of ethical implications and the development of processes that address these challenges. Concerns regarding the loss of human touch, authorship, and intellectual property arise with the role of AI in design (Adeleye, 2024). Ethical issues, such as transparency, fairness, and bias, must be addressed to ensure that AI-driven solutions uphold ethical standards (C. Y. Kim et al., 2024). It is essential to identify algorithmic biases, as AI logo makers often rely on algorithms that may not fully incorporate essential design principles such as proportion, balance, and unity, leading to inconsistent logo designs (Bertão et al., 2025). Additionally, the training data for AI models can favour specific languages and cultural contexts, resulting in outputs that favour Western-centric visual vocabularies (Kudless, 2023).

#### 2.1.2. The Role of Dynamic Logos in Consumer Engagement

The effectiveness of dynamic logos varies among consumer segments; thus, brands need to understand their target audience’s preferences and perceptions. Aligning dynamic elements with consumer expectations is crucial for reinforcing the brand’s premium positioning and for maximising its appeal (Vidal et al., 2016). Dynamic logos have a significant influence on consumer perception and brand recognition through various visual elements and their psychological effects. Research indicates that logos are critical components of corporate identity, affecting brand awareness, perception, and consumer behaviour. The following factors should be considered:

- Visual thickness: Thicker logos are perceived as being more powerful, enhancing the brand’s personality and consumer judgments (Eyni et al., 2023).
- Shape dynamics: The shape of a logo (upright vs. inclining) affects the perceived movement, reliability, and innovativeness, influencing product evaluations (X. Zhou et al., 2019).
- Dynamic imagery: Static logos can evoke a sense of movement, which increases consumer engagement and positively affects brand attitudes (Cian et al., 2014).
- Congruence with brand characteristics: The effectiveness of dynamic imagery is moderated by its alignment with the brand’s identity, enhancing consumer connection (Cian et al., 2014).

While dynamic visualisations offer flexibility, they can also introduce distortions. Therefore, it is imperative to assess their reliability to ensure that they accurately represent the intended data or message (Karinshak et al., 2023). Furthermore, the existing logo literature is fragmented and lacks a cohesive research framework; this review highlights gaps and limitations in the existing body of work. The wide range of topics discussed suggests a lack of depth or focus in some areas, indicating opportunities for further investigation (Z. Su et al., 2019). The findings of another notable study may not be directly generalisable to large-scale datasets, suggesting that the results could vary significantly when applied to more complex or expansive graph visualisations beyond the scope of the study (Federico & Miksch, 2016).

#### 2.1.3. The Relationship Between Logo Design, Brand Recognition, and Emotional Engagement

Emotional engagement is pivotal in the interplay between logo design and brand identity, as it shapes consumer perceptions and cultivates brand loyalty. An effective logo design functions not only as a visual identifier but also elicits emotional responses that resonate with the brand’s values and identity. This emotional connection can significantly enhance a brand’s reputation and consumer engagement. Emotional design is crucial for establishing a robust urban brand identity, as it addresses the emotional needs of consumers (J. Zhou, 2023). Logos that embody emotional characteristics can bolster corporate reputation, as evidenced by research on leading brands, which found that emotional states significantly influence stakeholder perceptions (İcil Tuncer et al., 2023). The evolution of logo design, particularly within libraries, underscores the importance of emotional expression and cultural significance, highlighting the significance of personalisation in brand identity (Tian & Song, 2024). The exclusive use of black-and-white logos, along with fictitious logos, brand names, and descriptions, may constrain a study’s capacity to capture existing consumer brand associations and real-world brand perceptions (Sharma & Varki, 2018). Another study encountered limitations in that while it provided a comprehensive analysis of visual elements by examining the top global brand logos, it did not consider potential variations in visual elements beyond the selected product categories or among lesser-known brands (Trehan & Kalro, 2024). The research indicates that the correlation between logo design and its constituent visual elements is considerably lower than anticipated, suggesting a limitation in the direct association between logo aesthetics and brand perception. Furthermore, it elucidates that while logos serve as corporate symbols encapsulating complex meanings, the depth of these associations may not be fully conveyed by the initial visual representation, thus revealing a constraint in comprehending the full scope of brand identity through logo design alone (César Machado et al., 2021). Emotional branding focuses on forming a connection between consumers and brands by evoking emotions. This strategy has shifted consumer behaviour from being product-centric to being emotion-centric, underscoring the importance of how brands make consumers feel (Jindal et al., 2022).

### 2.2. Development of Hypotheses

Dynamic logos, with their kinetic properties and visual salience, demonstrate high efficacy in capturing consumer attention. This enhanced attentiveness frequently correlates with increased consumer engagement with the brand (Jian et al., 2022; Yang et al., 2024). Dynamic elements in logos can elicit arousal, positively influencing purchase intentions. However, the impact of this stimulation varies based on the nature of the motion and consumers’ perception thereof (Fennell & Schneider, 2023). The prominence and design of logos, particularly their dynamic elements, play a pivotal role in shaping consumer perceptions of luxury brands. The emotional value evoked by these elements significantly impacts purchase intentions, underscoring the critical role of logo design in luxury brand marketing strategies (Butcher et al., 2016). The perception of luxury and elegance is frequently associated with dynamic elements, as they convey sophistication and modernity. Emotional content plays a vital role in consumer decision-making, enhancing the appeal and impact of such designs (Vaidya & Kalita, 2021). Luxury brands gain significant advantages by aligning dynamic logo elements with their consumers’ perceived self-congruence. This strategic alignment strengthens brand attachment, as consumers view the brand as a reflection of their actual or ideal self, ultimately fostering increased consumer advocacy (Shimul & Phau, 2022). Dynamic logos are designed to be flexible, enabling brands to adjust their visual identity in response to evolving market trends and consumer preferences. This adaptability is essential for sustaining brand relevance and recognition in premium segments, where innovation and exclusivity are highly valued (H. Su et al., 2020). Dynamic imagery in logos engages consumers with perceptual and conceptual fluency, fostering ease of processing and emotional resonance. This is especially vital in premium segments, where consumers prioritise brands that deliver memorable and immersive experiences (Miceli et al., 2014).

Consequently, the following hypotheses, designated as H1, H2, H3, and H4, are proposed:

**H1.**  *Logos with dynamic elements significantly enhance consumer attention compared with static logos, as measured by Start–End Attention, Total Attention, and Time Spent scores and Saliency Maps.*

Research has demonstrated that animated logos are more effective in capturing consumer interest, thereby enhancing engagement and purchase intentions. Animated logos utilise motion to capture attention, yielding higher Start–End Attention scores compared with static logos (Yang et al., 2024). Empirical evidence suggests that consumers allocate more time to viewing dynamic logos, which is associated with increased brand recall and recognition (Fennell & Schneider, 2023). Saliency maps indicate that dynamic elements more effectively capture visual attention, thereby emphasising key brand features and messages (M. Yu et al., 2024).

**H2.**  *The inclusion of dynamic elements in logos increases cognitive demand but generates higher emotional engagement and positivity compared with static logos, as measured by Cognitive Demand, Engagement, and Emotional scores.*

Metrics such as Total Attention and Time Spent demonstrate that dynamic logos maintain consumer engagement for extended periods, thereby enhancing brand recall and recognition (Yang et al., 2024). Furthermore, advanced saliency prediction models suggest that dynamic logos exhibit greater visual salience, resulting in higher attention scores compared with static logos (Hosseini et al., 2024).

**H3.**  *Logos with dynamic elements will result in higher brand industry awareness and alignment with premium market segments, as measured by Memory scores, Brand Perception metrics, and Industry Association metrics.*

The integration of dynamic elements within logos can substantially enhance brand recognition and foster customer loyalty across diverse market segments. Empirical evidence suggests that dynamic logos have varying impacts on hedonic and utilitarian products, resulting in improved market performance when appropriately aligned (Wang et al., 2023). Moreover, dynamic visual identities align with contemporary consumer preferences, augmenting their visual appeal and engagement, particularly among younger demographics (M. Yu et al., 2024). The perception of movement in logos, even when static, can evoke dynamic imagery that enhances consumer engagement and positively influences brand attitudes (Cian et al., 2014).

**H4.**  *A combined methodology of AI-powered eye tracking and qualitative cognitive surveys provides a more comprehensive assessment of logo performance by evaluating the results.*

Artificial-intelligence-powered systems enable real-time feedback on logo performance, facilitating prompt adjustments and optimisation. This capability is particularly valuable in fast-paced marketing environments that require rapid responses (Cazzato et al., 2020). Eye tracking captures where and how long a viewer looks at different logo elements, providing valuable data on visual attention and engagement. Metrics such as fixations and saccades can reveal which parts of a logo attract the most attention and how viewers visually navigate the logo (Maddox et al., 2018; Skaramagkas et al., 2023). Qualitative cognitive surveys provide flexibility in examining various cognitive functions and responses. They can be customised to target specific aspects of logo performance, such as visual appeal or memorability, enabling a more focused and tailored research approach (Clark & Maguire, 2020).

#### 2.2.1. Theoretical Foundation of the Hypotheses

The formulation of the study’s four hypotheses is grounded in interdisciplinary theoretical models from cognitive neuroscience, branding theory, and consumer psychology.

H1 is informed by the Dynamic Attention Theory, which posits that motion or perceived motion in visual stimuli naturally draws heightened attention due to their evolutionary salience and novelty (Wooley et al., 2022). When applied to logo design, this theory suggests that dynamic elements—especially those implying motion—will outperform static forms in capturing visual attention. Prior work indicates that moving or changeable design features trigger reflexive attentional shifts and extended fixation durations, both of which are measurable via eye-tracking metrics (Cian et al., 2014; Z. Liu, 2024).

H2 builds on the Arousal Theory (Duan, 2017; Fennell & Schneider, 2023; N. Kim & Lee, 2021) and the Cognitive Load Framework, which suggest that emotionally salient or novel stimuli can increase both mental processing effort and affective engagement. While increased complexity may challenge processing fluency, it can simultaneously amplify emotional resonance, especially in luxury contexts, where complexity often implies exclusivity. Therefore, logos that elicit a higher cognitive demand may also provoke stronger emotional reactions, as shown through EEG and implicit testing (Schettini et al., 2023; Vitolo et al., 2021).

H3 draws on the Self-Congruence Theory and Luxury Brand Identity Models, which emphasize the symbolic function of design elements in aligning with a consumer’s actual or ideal self-image (Shimul & Phau, 2022). When visual identity elements signal prestige, innovation, or expertise, they facilitate stronger consumer–brand identification. In this framework, dynamic logos serve not only as attention mechanisms but as carriers of meaning that enhance brand–industry alignment and premium positioning (Butcher et al., 2016; Wang et al., 2023).

H4 reflects current methodological perspectives in neurodesign and consumer neuroscience, which advocate the integration of AI-based neurometric prediction with traditional qualitative and cognitive measures (Clark & Maguire, 2020; Ramsøy, 2019; Zoëga Ramsoy et al., 2023). A hybrid approach allows for a more complete assessment of subconscious responses (e.g., through predictive EEG, memory, and attention data) and conscious evaluations (e.g., emotional survey data), yielding a holistic understanding of consumer perception and behaviour in branding contexts.

#### 2.2.2. Research Gap

Research utilising eye-tracking technology to examine dynamic and static logos has yielded significant insights, yet several research gaps persist. While existing studies have demonstrated that static logos can evoke dynamic imagery, thereby influencing consumer engagement and attitudes, the specific effects of dynamic logos on attention and memory remain underexplored. This response outlines key areas that necessitate further investigation.

##### Gaps in Dynamic Logo Research

Comparative Analysis: There is a paucity of direct comparisons between static and dynamic logos concerning eye-tracking metrics, such as fixation duration and frequency, which could elucidate differences in consumer engagement (Cian et al., 2014).

Dynamic Attention Theory: The application of Dynamic Attention Theory to logo design is somewhat limited. Although research has shown that dynamic stimuli can alter attention patterns, this has not been extensively applied to logo studies (Wooley et al., 2022).

Longitudinal Studies: Most existing research focuses on immediate responses to interventions. Longitudinal studies could provide insights into how perceptions of logos evolve with repeated exposure to dynamic versus static designs (Boettcher & Nobre, 2025).

##### Methodological Limitations

Contextual Variability: Current studies often take place in controlled environments, which may not accurately reflect real-world interactions with logos. More representative settings could yield different engagement patterns (Fitton Davies et al., 2024).

Integration of User Behaviour: The integration of eye tracking with user logging in dynamic contexts remains underexplored. This integration could enhance our understanding of how users interact with logos in interactive environments (Ooms et al., 2015).

##### Limited Exploration of Eye Tracking in Real-World Contexts

Most eye-tracking studies are conducted in controlled environments, which may not accurately reflect real-world consumer interactions with logos. Fitton Davies et al. emphasise the importance of conducting eye-tracking research in more representative settings, such as dynamic sports contexts, to better understand visual attention in natural environments (Fitton Davies et al., 2024). The integration of eye tracking with user logging, as suggested by Ooms et al., could provide deeper insights into how consumers interact with dynamic logos in interactive applications (Ooms et al., 2015).

##### Challenges for Eye-Tracking Technology When Using Dynamic Stimuli

The accuracy of eye-tracking devices in dynamic conditions remains a challenge. Onkhar et al. found that head-mounted eye-trackers like Tobii Pro Glasses have varying accuracy depending on the dynamicity of the task (Onkhar et al., 2023). Drews and Dierkes propose enhancements to fixation detection algorithms for dynamic scenarios, which could improve the reliability of eye-tracking data in studies involving dynamic logos (Drews & Dierkes, 2024).

While existing research studies have provided valuable insights into the perception of dynamic imagery and visual attention, there is a need for more comprehensive studies that address the limitations of existing methodologies. Exploring the use of advanced eye-tracking technologies, as presented in this study, and conducting research incorporating high-end AI neuroscientific technology, as utilised in this research, could significantly enhance the understanding of how dynamic and static logos influence consumer behaviour.

## 3. Materials and Methods

The study utilised diverse sample sizes for various components of the research:

1. AI-powered eye tracking: *n* = 255,000
2. EEG technology: *n* = 45,000
3. Implicit testing: *n* = 9000
4. Memory testing: *n* = 7000
5. Qualitative cognitive surveys: *n* = 297

Following data cleaning, the final sample for the qualitative cognitive surveys consisted of 113 female respondents. The study specifically targeted female participants in the United States, aged 20–40, who had purchased cosmetics within the past year and who reported an annual income of USD 50,000 to USD 99,000.

The rationale for the sample sizes:

1. AI-powered methods: The substantial sample sizes (255,000 for eye tracking, 45,000 for EEG) were employed to train and validate the AI models, thereby ensuring robust predictions of human behaviour.
2. Implicit and memory testing: Moderately large samples (9000 and 7000, respectively) were utilised to capture subconscious responses and memory retention.
3. Qualitative cognitive surveys: The initial sample of 297 participants was selected to balance statistical power with the practical constraints of administering the survey. After data cleaning, the final sample of 113 female respondents was considered adequate for the following reasons:
  - Targeted demographic: The study focused on a specific consumer segment (female, US-based, 20–40 years old, recent cosmetics purchasers, with an income of USD 50,000–USD 99,000), resulting in a more homogeneous sample.
  - Alignment with luxury cosmetics consumers: The sample profile corresponded with the target demographic for luxury cosmetics brands.
  - Balancing depth and breadth: The sample size permitted an in-depth analysis of emotional responses and industry associations while maintaining practical feasibility.
  - Complementary to AI-driven data: The qualitative surveys were designed to supplement the large-scale AI-predicted data, providing context and validation rather than serving as the primary data source.

The integration of large-scale AI predictions and targeted qualitative surveys was aimed at delivering a comprehensive and representative analysis of consumer responses to logo designs in the luxury cosmetics sector.

**Figure A1:**
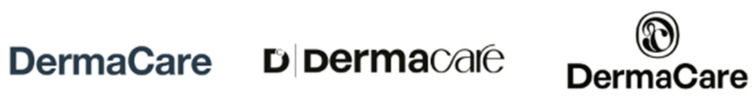
Logos utilised in this research study–Logo 1 (**left**), Logo 2 (**centre**), and Logo 3 (**right**).

The study employed three distinct logo designs to compare static and dynamic elements in branding systematically: Logo 1, the current logo used by the retailer in commercial business settings, which features a simple design with the verbal trademark “DermaCare” in Helvetica Neue Pro Bold font; Logo 2, a newly designed version for this research that introduces a dynamic element at the end of the word “Care” and utilises a combination of General Sans, Montserrat, and Loving Ambros fonts; and Logo 3, which combines a design similar to Logo 1 with a dynamic icon placed above the brand name and uses General Sans and Montserrat fonts (see Figure A1 for visual representations). Professional brand designers created these logos specifically for this investigation, enabling controlled comparisons and minimising confounding variables. For this research, we decided to utilise a luxury retail logo for neuromarketing testing due to the following factors: (a) DermaCare is a prominent distributor of luxury brands in Southeast Europe, representing premium products from brands such as Dermalogica, Jane Iredale, and Advanced Nutrition. Studies indicate that emotion-based advertisements can have a profound influence on consumer decision-making, underscoring the importance of emotional engagement in luxury branding (Alsharif & Isa, 2025); (b) The brand’s verbal trademark offers several advantages for marketing and branding. It enables multisensory stimulation by combining verbal and visual engagements, involving both auditory and visual processing. This enables the precise measurement of which brand elements may lead to stronger neural responses and memory formation. Eye tracking complements EEG by visualising consumer attention patterns, showing that endorsements in advertisements significantly enhance viewer preferences (Mashrur et al., 2024). Studies show that luxury logos lead to smaller N2 amplitudes, indicating reduced cognitive conflicts and more considerable Late Positive Potentials (LPP), reflecting heightened emotional arousal. This suggests that luxury logos enhance memory recall by minimising cognitive dissonance while maximising emotional engagement (Peng et al., 2020); (c) The verbal trademark also enhances cognitive processing and emotional resonance. The integration of text and images may require an increased cognitive effort, while icons can evoke rapid emotional responses. The verbal element provides context and reinforces the intended message. Additionally, a unique combination of verbal and visual elements can help distinguish the brand in a crowded marketplace, potentially increasing industry awareness. This multimodal approach, combining EEG and eye-tracking data, can inform luxury brands about effective advertising strategies, thereby enhancing their market positioning (Georgiadis et al., 2023; Audrin et al., 2017); (d) The verbal trademark provides versatility in testing, cultural adaptability, and enhanced brand recall. Using both verbal and visual elements allows for the separate testing of each component and their combined effect, providing comprehensive insights. Icons can transcend language barriers, while verbal elements can be tailored to specific markets, allowing for broader applicability in global neuromarketing studies. Finally, the combination of verbal and visual elements may create stronger memory associations, enhancing overall brand recall. The dorsolateral and ventromedial prefrontal cortex, along with the orbitofrontal cortex, are activated when individuals process luxury brand labels. These areas are associated with reward processing and decision-making, indicating that luxury logos may enhance memory through their emotional and motivational significance (Audrin et al., 2017). Both emotional survey testing (conducted with 297 participants from the US market) and subliminal testing methodologies (utilising AI-powered eye tracking and EEG technologies to predict subconscious attention patterns and engagement levels, offering insights beyond traditional self-reporting methods) were employed to assess the research questions comprehensively. This multi-method approach captures both implicit and explicit consumer responses, providing a comprehensive understanding of the effectiveness of logos. The integration of AI predictions with human surveys enables the validation and cross-referencing of results, thereby enhancing the robustness and reliability of the findings.

### 3.1. AI-Driven Neuroscience Methods Setup

The three logos were placed on a white background and saved as an image with a resolution of 3240 × 3240 pixels. Several metric sets from this software were utilised, including EEG (electroencephalograph), eye tracking (refer to description (a)), memory testing (refer to description (b)), and Fast Response Test (FRT) metrics. Specific Areas of Interest (AOIs) were identified for each design, encompassing the entire logo and its constituent elements, including the primary word (“Derma”), secondary word (“Care”), and accompanying iconography (present in logo designs 2 and 3). Metrics such as Engagement, Time Spent, Total Attention, Start and End Attention, Cognitive Demand, and Memory were quantified as percentages to assess whether participants responded more swiftly, engaged more thoroughly, and formed stronger associations with the industry brand in logos featuring dynamic iconography. Slope graphs, heat maps, and tables were employed to compare these metrics. Additionally, saliency maps were generated to capture participants’ fixation points, providing actionable insights into consumer behaviour and logo performance. Correlation analysis was conducted to evaluate the relationships among the metrics and to assess differences among the logo fixation points. Data visualisation, correlation analysis, and saliency mapping were performed using Google Colab with Python version 3.10.12. The libraries utilised in this analysis included TensorFlow, Pandas, NumPy, Matplotlib, and Plotly Express. The neuroscientific software Predict (version 1.0) from Neurons Inc. (Copenhagen, Denmark) was utilised for the subconscious testing. This software employs an artificial intelligence eye-tracking predictive model trained on a substantial dataset of *n* = 250,000 participants and recorded with an eye tracker (Eye Tracker: Tobii X2-30, Tobii Pro AB, Danderyd, Sweden) in collaboration with Stanford University and AI EEG recordings (*n* = 45,000), which both predict human behaviour based on prescreen recordings as described. A study comprising 30 participants was used to classify feature-based attention using single-trial EEG data. This sample size was deemed sufficient for developing and benchmarking algorithms in this context, suggesting that 30 participants can be adequate for certain EEG studies focused on specific neural mechanisms (Renton et al., 2022). The precision and accuracy of eye-tracking technology can significantly influence the required sample size. Investigations utilising advanced technologies may achieve reliable results with smaller samples due to their enhanced data quality (Doherty et al., 2010). AI-driven insights into consumer behaviour can inform brand management and marketing strategies. By understanding sensory, emotional, and personalised experiences, brands can adjust their strategies to meet better consumer needs and preferences (Luo, 2024). Eye-tracking technology, as utilised in neuromarketing, facilitates the identification of elements in advertisements that capture consumer attention (Neurons, 2024b). This method reveals that novelty, rather than colour, is a primary driver of attention in advertising, which can be crucial for luxury brands aiming to differentiate themselves in a saturated market (Fondevila-Gascón et al., 2023). EEG measures, primarily frontal alpha asymmetry, are reliable indicators of consumer preferences and emotional responses to marketing stimuli. AI-powered eye-tracking and EEG technologies provide objective measures of subconscious attention patterns and engagement levels, offering insights that surpass those provided by traditional self-reporting methods. Personalised persuasion through AI, such as using large language models (LLMs) to match marketing messages to consumer psychological profiles, can significantly enhance the effectiveness of marketing campaigns (Matz et al., 2024). The Predict software’s extensive dataset, comprising 100 billion data points, enables highly accurate predictions of human visual attention, surpassing the limitations of conventional survey methodologies. These measures can differentiate between positive and negative consumer responses, providing luxury brands with insights into the perception of their advertisements (Byrne et al., 2022). All participants were pretested on tens of thousands of assets, where 85% of the data were from online testing, with half of these utilising webcam eye tracking (these data have a lower temporal resolution but still yield approximately 15,000 data points per person, providing a database of over 3.8 million raw data points) (Neurons, 2024a, 2024b).

(a) For eye tracking and EEG recordings, the database has a global representation: USA (35%), UK (20%), Nordic countries (20%), the DACH countries (10%), Southern Europe (5%), Latin America (3%), Middle East (3%), Asia (2%), and Southeast Asia (2%); an age range of 18–55; and a gender split of 50:50.
(b) Memory metrics were also part of the Predict software, but the recordings were conducted on a different sample size (*n* = 7000), with an age range of 15–55 and a 50:50 gender split of English speakers from the US and UK (Ramsøy, 2025). Research on memory has demonstrated that specific visual attributes can serve as predictors of memorability, which may be applied to luxury brand logos to enhance their recognition and recall by emphasising distinctive perceptual characteristics (Davis & Bainbridge, 2023). Ethical requirements were fulfilled as all tested subjects provided prior consent for their data to be used in the AI algorithm. The software’s responses enable highly accurate (95% accuracy) predictions of human visual attention compared with high-precision eye-tracking (Zoëga Ramsøy, 2023a). The Memory AI algorithm employs a data-driven approach derived from the Visual Memory Game methodology developed by MIT (Khosla et al., 2015). The datasets used to train the model encompass various advertising materials, capturing diverse industry-use cases—from print and out-of-home (OOH) displays to digital platforms such as websites and social media. This dataset is continuously enriched to ensure that the model consistently captures the latest participant behaviour and trends in advertising (Neurons HQ, 2023; Zoëga Ramsøy, 2023b).
(c) The Fast Response Test (FRT) prediction model is also part of the same software, and the metrics we used in our analysis measure positive emotional responses to media content. In the context of luxury logo testing, implicit measures can reveal underlying attitudes towards a brand that are not accessible through explicit questioning. This may encompass associations with prestige, quality, or exclusivity integral to luxury branding (Charlesworth et al., 2022). With these metrics, engagement levels can be predicted with an accuracy of 92% on test datasets (Neurons, 2024a, 2024b). This robust statistical foundation enables a confident estimation of how effectively an advertisement will emotionally engage its audience based on the initial exposure. For FRT-based metrics, the software provider required a minimum of 100 participants to obtain a reliable measure of asset responses, confirming that we had a highly reliable AI set of metrics for this research. The FRT methodology involved participants aged 18–55, with an equal male–female ratio (50:50) (*n* > 9000) from geographical locations including the EU, USA, and UK. Participants viewed the content for a few seconds and subsequently rated it by selecting “yes” or “no” to association words, such as “Engaging”, “Interesting”, and “Happy”. Their response time was also recorded, lending weight to the intensity of their sentiment. The provider ensured that the FRT score was comparable and reliable by normalising the response times and responses across multiple participants. This prediction model utilises the EfficientNetB2V2 architecture, renowned for its efficiency in computer vision tasks. With approximately 8.8 million trainable parameters, the model demonstrates proficiency in connecting visual inputs to engagement scores derived from meticulous regression analyses. The model resizes visuals to maintain aspect ratios and applies GradCAM to identify impactful image features for predictions (Ramsøy, 2019). Luxury brands can leverage AI-predicted human behaviour to create targeted marketing campaigns and increase sales by utilising advanced AI technologies to personalise consumer experiences and optimise marketing strategies. AI tools, such as convolutional neural networks (CNNs), AI-powered try-on technology (ATT), and generative AI models, can analyse consumer behaviour and predict preferences, enabling brands to tailor their marketing efforts more effectively. This approach not only enhances customer satisfaction but also drives business model innovation in the luxury sector (Alipour et al., 2024; Hermann & Puntoni, 2024; Madanchian, 2024; Matz et al., 2024).

### 3.2. Emotional Survey Methods Setup

**Figure A2:**
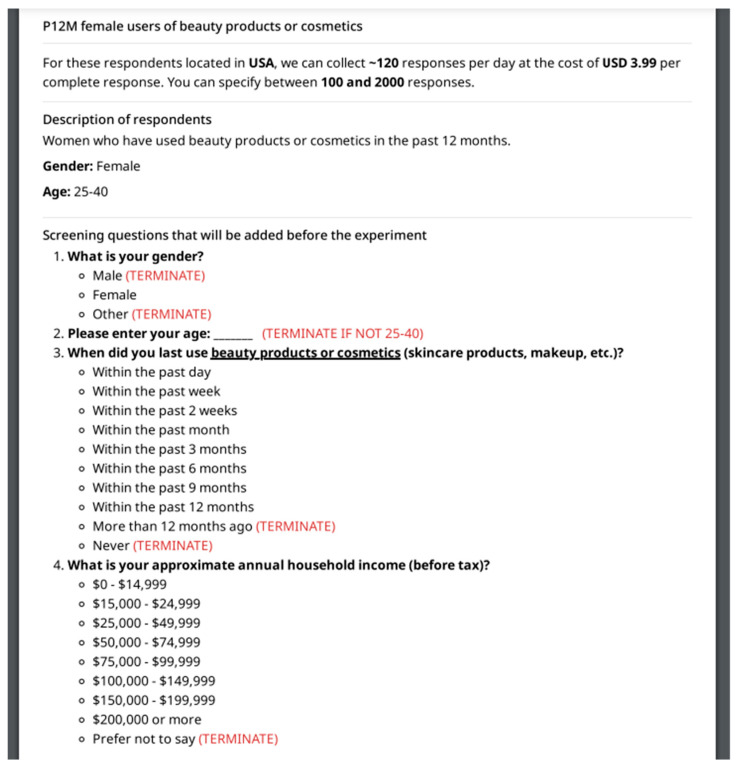
Preselection survey used for cognitive testing.

**Figure A3:**
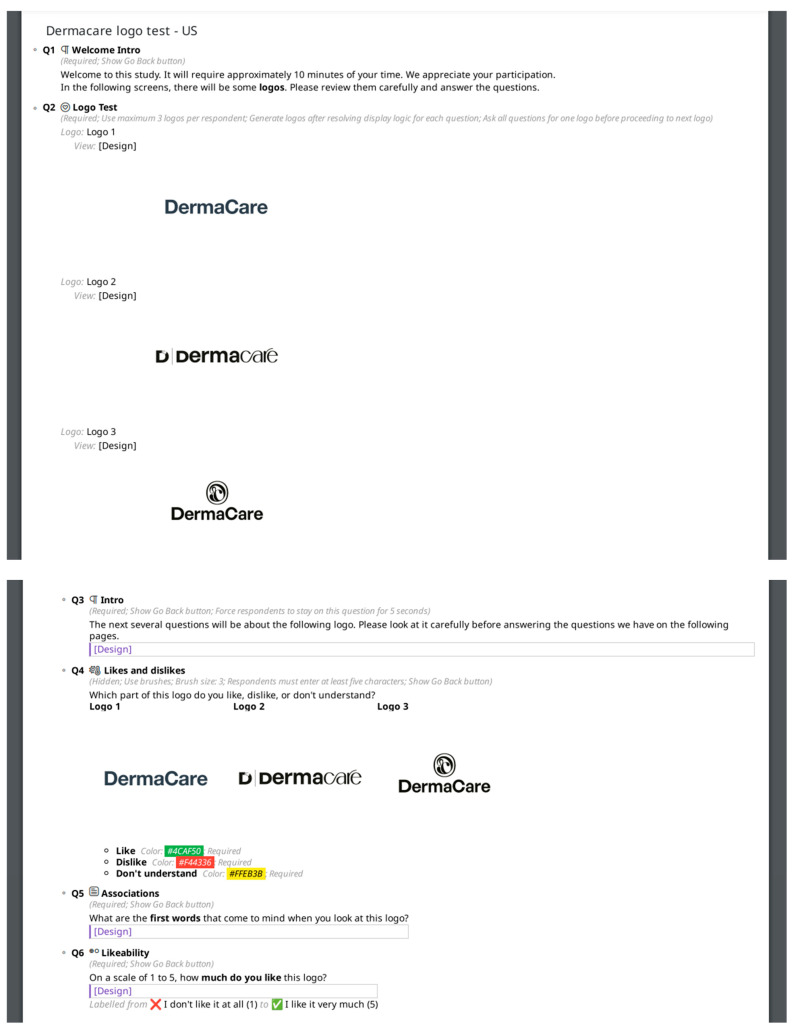

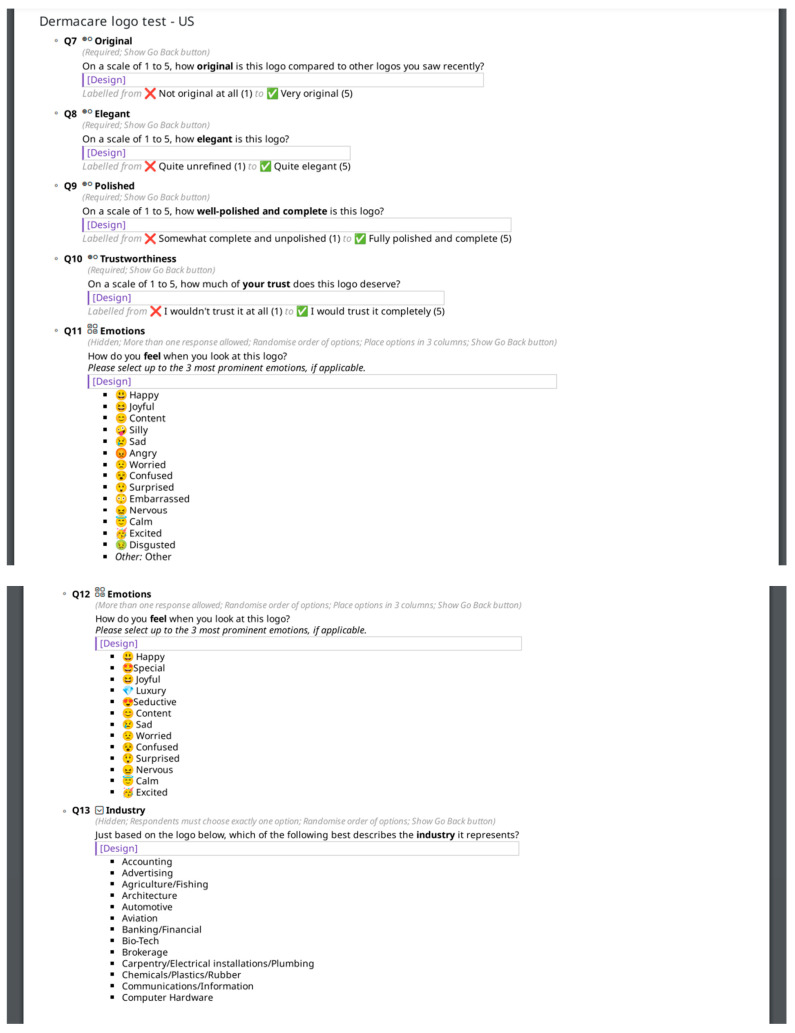

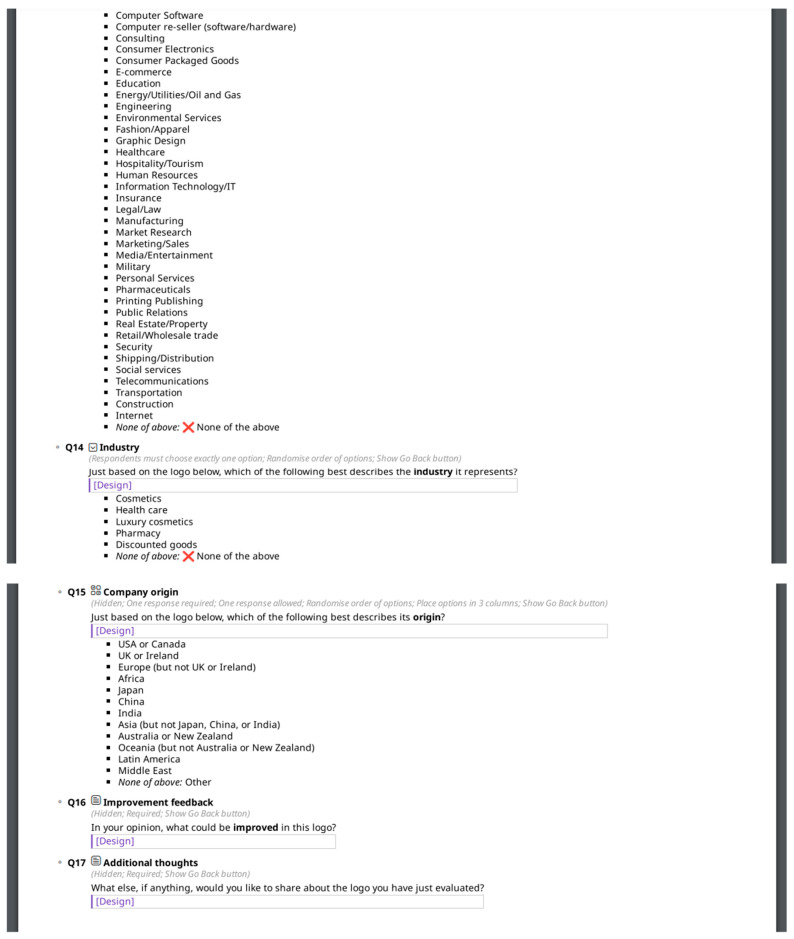
Questionnaire design and implementation for cognitive testing.

Survey research is vulnerable to various biases that can significantly affect data accuracy. These biases arise from methodological flaws, participant demographics, and the characteristics of survey instruments (Shahbazi et al., 2023; Weber et al., 2021; Westland, 2022). To mitigate these biases, an online survey was conducted to assess emotional responses and industry associations using the Conjointly software platform (*n* = 297), which specialises in conjoint analysis, and utilises its database panel. Nonresponse bias is intensified by the mode of survey administration, with mixed-mode surveys often resulting in less bias compared with face-to-face methods (Rybak, 2023). To mitigate nonresponse bias in this study, the panel was recruited and provided with financial compensation. All testing was conducted on the Institute for Neuromarketing and Intellectual Property premises. The Institute for Neuromarketing and IP’s ethics committee approved this research (Ref: IRBJINIP2024-2) and supervised the study to ensure compliance with local and international ethical guidelines. Participants’ data were processed by the GDPR (General Data Protection Regulation) and the European Code of Ethics for Research. Following data cleaning to exclude incomplete survey responses, the final sample comprised 113 female respondents. The study targeted female participants from the United States, aged 20–40, who had purchased cosmetics within the past year and who reported an annual income of USD 50,000–USD 99,000, aligning with the demographic profile of luxury cosmetics consumers. The United States market is characterised by a pronounced cultural predisposition towards prestige and luxury brands, which is influenced by social status, self-expression, and perceived quality. This cultural context provides an optimal environment for luxury brands to explore consumer motivations and preferences (Sung et al., 2020). A preselection survey (Figure A2) was conducted to ensure that participants met the age, location, income, and purchasing behaviour criteria. The significance of moral emotions in consumer responses to brand activism underscores the necessity of aligning luxury brand communications with consumer values to elicit favourable emotional reactions (Wannow et al., 2024). Only those who satisfied these requirements could proceed to the primary survey. The survey instrument consisted of 14 open-ended questions with a combination of Likert Scale testing (1–5) and multiple-choice options (see Figure A3) that were designed to measure 13 distinct emotions associated with luxury brands (happiness, feeling special, joyfulness, luxury, seductiveness, contentment, sadness, worry, confusion, surprise, nervousness, calmness, and excitement) across 49 different industries (see Figure A3). This approach assessed participants’ initial associations with logos and industries, as well as their emotional responses. Participants evaluated each logo on a 5-point Likert scale, ranging from 1 (“I do not like it at all”) to 5 (“I like it very much”), based on brand perception metrics that included likeability, originality, elegance, polish, and trustworthiness. Likert scales are frequently employed in diverse fields, including luxury branding, to quantify consumer responses. These scales demonstrate efficacy in measuring consumer attitudes towards luxury brand logos, which are often associated with emotional and symbolic consumption (Morando & Platania, 2022; Pescaroli et al., 2020). The survey also captured emotions evoked by the logos, encompassing general emotions (happiness, joyfulness, contentment, sadness, worry, confusion, surprise, nervousness, calmness, and excitement) and emotions specific to luxury brand identity (luxury, seductiveness, and feeling special). Furthermore, respondents’ associations of the logos with relevant industries, such as cosmetics, healthcare, luxury cosmetics, pharmacy, and discounted goods, were assessed. The physiological and psychological dimensions of emotions necessitate sophisticated tools and methodologies to measure and interpret these variations accurately (Fu et al., 2022). Understanding these complexities is crucial for developing effective emotional regulation strategies and interventions in both personal and societal contexts. The complete survey trial required 10 min per participant. The post-survey analysis involved calculating the top-two box score (the sum of percentages for ratings 4 and 5) to determine the proportion of respondents who provided the highest and second-highest ratings. Various data visualisation techniques were employed to compare emotional performance and brand industry associations among the three logos. These included horizontal bar charts, radar charts, slope graphs, and stacked bar charts, which were all generated using Python 3.10.12 and executed in Jupyter Notebook 6.5.5. The analysis utilised the Pandas, NumPy, Matplotlib, and Plotly Express libraries. These visualisations effectively highlighted the differences among the three logos, particularly emphasising the impact of incorporating dynamic elements on emotional metrics.

## 4. Results

This section presents the study’s findings on the impact of dynamic logo elements on consumer engagement, emotional connection, and brand recall. The results are organised by the hypotheses and offer a comprehensive analysis of the data obtained through AI-powered eye tracking, EEG, and qualitative surveys.

### 4.1. Comparative Analysis of Start–End Attention Scores: Evaluating Logo Design Effectiveness

**Figure 1:**
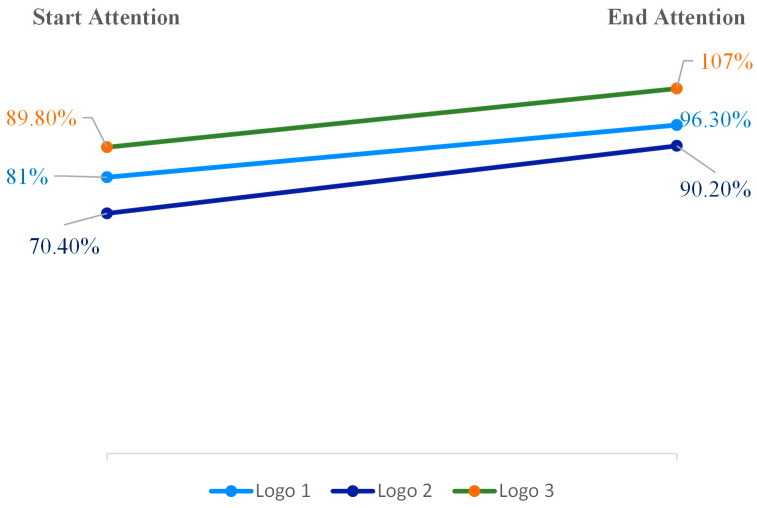
Comparative analysis of the initial and final attention distribution across the three logo designs.

**Figure A4:**
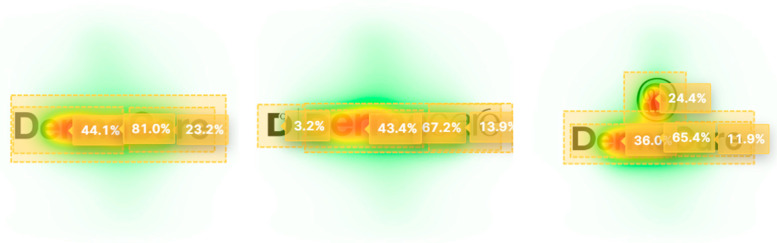
Start attention–Logo 1 (**left**), Logo 2 (**middle**), and Logo 3 (**right**).

**Figure A5:**
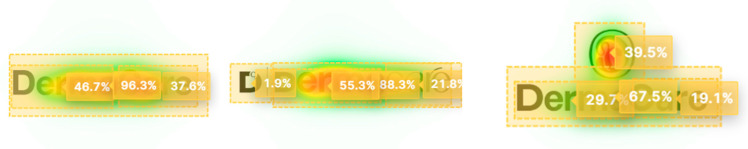
End attention–Logo 1 (**left**), Logo 2 (**middle**), and Logo 3 (**right**).

To assess the effectiveness of logo design, the Start–End Attention scores for each logo were analysed. The Start Attention metric measures the initial appeal of the content within the first two seconds, whereas the End Attention metric evaluates the sustained appeal during the final two seconds. To determine the total Start and End Attention scores for each logo, all AOI scores within the logo, including the logo icon score and the logo text score, were aggregated. The results for the Start Attention and End Attention AOIs are presented in Figure A4 and Figure A5. For example, the End Attention score for Logo 3 (107%) was derived from the sum of the logo icon score (39.5%) and the logo whole-text score (67.5%). The 100% benchmark indicates the expected performance for an attention-grabbing logo as measured by the End Attention metric. This metric is crucial for understanding how quickly and effectively content can engage viewers, which is particularly relevant in digital environments where attention spans are short. The research highlights the importance of the initial engagement, which can be influenced by various factors, including temporal landmarks, content type, and presentation (Bi et al., 2021). End Attention captures the exploratory top–down attention, revealing elements that maintain viewer engagement until the conclusion (Neurons, 2024c). Attentional weights are influenced by both spatial location and non-spatial features, such as colour or shape, which interact multiplicatively to determine the initial capture of attention (Nordfang et al., 2018). To compare this metric, a slope graph will be utilised to evaluate the retention of customer attention for each logo. Our findings elucidate the progression of attention from initiation to conclusion across the three logo designs (see Figure 1). Logo 3 demonstrated the most robust performance, starting with 89.8% attention and increasing substantially to 107% by the end, showcasing its ability to capture, retain, and amplify viewer attention over time.

In this study, percentages exceeding 100% are feasible and denote exceptionally high levels of attention. The research employs a proprietary AI-driven eye-tracking predictive model that normalises data across multiple participants. This normalisation process permits scores surpassing 100%, which signifies attention levels substantially above the average or baseline. This suggests that the dynamic icon and design of Logo 3 are highly efficacious in sustaining engagement. Logo 1 initiates at 81% attention and rises to 96.3%, indicating a steady improvement in attention retention. While it does not perform as strongly as Logo 3, it still maintains a substantial viewer focus due to its simplicity. Logo 2, conversely, begins with the lowest attention at 70.4% and concludes at 90.2%, showing an improvement but still underperforming compared with the other two logos. Dynamic logotypes should strive to achieve an equilibrium that facilitates initial engagement through visual intricacy while ensuring the use of conceptual elements that enhance their appeal over time (Miceli et al., 2014). Despite the dynamic element in the word “Care”, it does not elicit the same level of attention retention as Logo 3, suggesting that not all logos with dynamic elements enhanced customers’ attention (H1 partially accepted). The findings partially corroborate Hypothesis 1, as not all logos incorporating dynamic elements enhanced customer attention equally. Specifically, the dynamic icon in Logo 3 proved remarkably effective, whereas the dynamic element in the text of Logo 2 did not achieve the same level of attention retention. The differences in attention scores among the three logo designs are statistically significant, as indicated by the *p*-values presented in Table 5. The correlation values for Logo 2 versus Logo 1 (0.402; *p* = 0.000 × 10^0^) and Logo 3 versus Logo 1 (0.155; *p* = 1.536 × 10^−27^) are both statistically significant. These low *p*-values suggest that the observed differences in attention distribution are unlikely to have occurred by chance, indicating that variations in logo design elements have a significant impact on viewer attention patterns.

### 4.2. Comparative Analysis of Attention Distribution Across the Logo Designs

**Figure 2:**
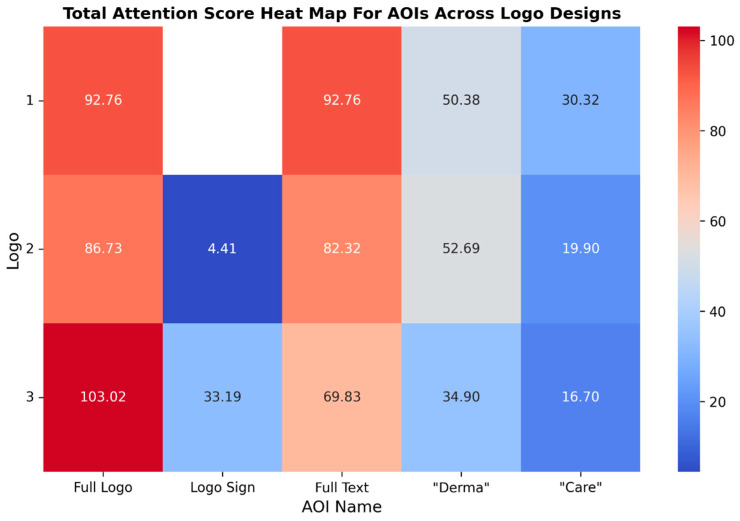
Comparative analysis of Total Attention across the three logos.

To discern the significance of key content elements and to clarify the viewer attention patterns, an analysis of the Total Attention metric was conducted. The Total Attention metric in this research highlights the salience of key content elements and elucidates viewer attention patterns, thereby emphasising the primary focal points of the logo. It has surpassed unsupervised saliency models in predicting fixations during free viewing, underscoring its significance in attention measurement (Chakraborty et al., 2022). The overall score for Total Attention is derived from our proprietary Total Attention heatmap algorithm. This algorithm processes the raw attention data, normalises it across different viewer demographics, and maps it onto the tested asset’s Areas of Interest (AOIs). Figure 2 illustrates the total attention distribution across the three logo designs, emphasising their overall visual attraction. Utilising a heat map provides an immediate colour-coded representation of the attention distribution, facilitating the identification of the most visually engaging elements within each logo design. This visualisation is consistent with neuromarketing principles of rapid cognitive processing, enabling readers to quickly pinpoint “hot spots” of high attention and cooler areas requiring enhancement. Furthermore, the intuitive format of the heat map facilitates data-driven decision-making, enabling readers to efficiently interpret complex attention metrics without requiring extensive statistical expertise. Logo 1, which lacks an icon, drew substantial attention to the “Derma” text (50.38%), followed by the “Care” text (30.32%). The total attention of Logo 1 was considered significant at 92.76%. However, the absence of an icon resulted in limited attention to the other elements, rendering the focus predominantly text-centric. A visual hierarchy initially directs users’ attention to the most salient elements, ensuring that critical information is perceived and processed efficiently. This is accomplished by strategically utilising the size, contrast, and placement of textual and other elements (She et al., 2021). Logo 2, featuring a small “DC” icon, demonstrated a slightly more dispersed attention distribution, with 4.41% of the attention directed to the sub-icon. However, the majority of the focus remained on the “Derma” text (52.69%) and the “Care” text (19.90%). The sub-icon attention score and the whole text attention score were aggregated into an entire logo’s attention score of 86.73%, indicating its inferior performance compared with Logo 1. In contrast, Logo 3, which incorporates a dynamic and visually prominent icon, exhibited a balanced allocation of attention.

The icon captured 33.19% of the focus, while the “Derma” (34.90%) and “Care” (16.70%) texts received attention. The observed variations in attention distribution across the three logo designs can be ascribed to several factors:

1. Visual complexity: Logo 3, characterised by its dynamic icon, demonstrates a more balanced attention distribution (icon: 33.19%; “Derma”: 34.90%; “Care”: 16.70%) in comparison with Logo 1 and Logo 2. This suggests that incorporating a visually distinct element enhances overall engagement by providing multiple focal points.
2. Element placement: The strategic positioning of the icon above the text in Logo 3 appears to effectively redistribute attention, whereas the text-only design of Logo 1 predominantly focuses attention on the word “Derma” (50.38%).
3. Dynamic versus static elements: The dynamic icon in Logo 3 appears to capture attention more effectively than the subtle dynamic element in the “Care” text of Logo 2, suggesting that the nature and prominence of dynamic features significantly influence attention allocation. The total attention of the entire logo was 103.02%, indicating a robust overall attention level. This suggests that the icon effectively redistributed attention across all elements and resulted in high customer attention. Icons can utilise co-saliency detection to guide user attention towards everyday salient objects within a group of images. This methodology reduces visual interference from non-essential elements, enabling users to focus more effectively on key objects (Ren et al., 2021). However, the data also indicate that not all logos with dynamic elements enhance customer attention (H1 partially accepted). The findings offer partial support for Hypothesis 1, as the logo featuring the most prominent dynamic element (Logo 3) exhibited the highest overall attention score. Nevertheless, the results also suggest that not all dynamic elements equally enhance customer attention.

The Time Spent metric utilises a refined attention-tracking algorithm to model the duration of attention paid to the AOIs. It captures the duration of attention over a 5 s exposure window, tracking moment-to-moment fluctuations in the gaze. The cumulative time spent on each AOI is then converted into a score, indicating the staying power of individual elements. The Time-Distributed Attention Network (TD-Atten) proposed by a study on EEG-based motor imagery decoding utilises attention mechanisms to assign weights to different input features, effectively quantifying the time allocated to processing these features. This approach underscores the significance of adaptive attention allocation in enhancing task performance (Ma et al., 2022).

The Time Spent comparative analysis employs a sophisticated attention-tracking algorithm to quantify consumer focus on Areas of Interest (AOIs). This method measures the attentional duration within a 5 s exposure period and monitors instantaneous gaze shifts. The aggregate time allocated to each AOI is subsequently translated into a numerical value that reflects the retention potential of specific elements. Table 1 provides a comparative analysis of the viewer attention patterns across the three logo designs, emphasising the allocation of time to various components. Logo 1, which comprises solely textual elements, exhibited a total viewing duration of 2.29 s, with 1.41 s dedicated to “Derma” and 0.88 s to “Care”. This distribution implies that the absence of visual elements prompted viewers to focus on the text. The analysis, conducted over a 5 s viewing period, utilises gaze tracking to monitor instantaneous shifts in viewer focus. The algorithm quantifies the total time spent on each Area of Interest (AOI), offering numerical values that reflect the retention potential of specific elements. Key components of this analysis include the following: 1. Gaze tracking: The algorithm monitors instantaneous shifts in the viewer’s gaze. 2. Quantification: The total time spent observing each Area of Interest (AOI) is calculated and converted into a numerical value. 3. Retention potential: These numerical values indicate the efficacy of specific elements in capturing and maintaining viewer attention. The analysis of Logo 1 indicates that the absence of visual elements led viewers to concentrate more on the text components. This suggests that when confronted with text-only logos, consumers tend to allocate more time to reading and processing the words. This process entails the construction and simplification of logical representations, as observed in nonverbal deduction tasks wherein individuals utilise cognitive inference to interpret incomplete information (Martín-Salguero et al., 2023). Logo 2, incorporating a subtle “DC” sub-logo, displayed a more equitable temporal distribution. The “Derma” and “Care” texts garnered 1.41 and 0.6 s of attention, respectively, whilst the sub-logo icon attracted a mere 0.18 s of attention, indicating its understated visual presence. The cumulative viewing time for Logo 2, comprising the sub-logo icon (0.18 s) and the “DermaCare” text (2.33 s), totalled 2.51 s—marginally less than for Logo 1. This outcome suggests that a subtle icon may not effectively enhance overall design engagement. This suggests that visual elements can play a significant role in communication; however, their efficacy is contingent upon the audience’s perception and utilisation (Zhang et al., 2022). Conversely, Logo 3 distinguished itself with a dynamic, visually prominent icon, significantly altering the distribution of viewer attention. The icon commanded 0.89 s of focus, substantially surpassing the sub-logo in Logo 2. Moreover, the “Derma” (0.93 s) and “Care” (0.49 s) texts received balanced attention, indicating that the icon enhanced rather than diminished textual engagement. The combined viewing time for the icon (0.89 s) and the brand name “DermaCare” (2.01 s) yielded a total of 2.9 s for Logo 3, rendering it the most effective among the three designs. In this analysis, Logo 3, featuring its distinctive icon, maintained the most extended duration of consumer attention, closely followed by Logo 1. While Logo 2, with its dynamic element in the word “Care”, performed marginally worse than its counterparts, the disparity was negligible—lagging behind Logo 1 by a mere 0.29 s and Logo 3 by 0.39 s (H1 partially supported). Luxury brands are advised to prioritise innovative solutions and resource allocation to enhance online customer engagement, indicating that more explicit and strategic branding efforts are necessary to maintain their market position (Hoang et al., 2022).

### 4.3. Comparative Analysis of Engagement, Cognitive Demand, and Memory Scores for Different Logo Designs

The interaction between Cognitive Demand and Engagement plays a crucial role in elucidating the influence of logo designs on viewer perception. Cognitive Demand reflects the mental effort required to process a logo, determining whether it enhances clarity or introduces complexity. Research indicates that heightened cognitive demands may lead to enhanced processing efficiency, as evidenced by the observed correlation between improved performance and increased selectivity within the multiple-demand (MD) network (Schettini et al., 2023). This section examines the Pearson Correlation between Cognitive Demand and Engagement across the three logo designs and supports this by scatter-plot visualisations. Analysing these relationships gives insights into how static and dynamic elements affect viewer engagement and cognitive processing.

**Figure 3:**
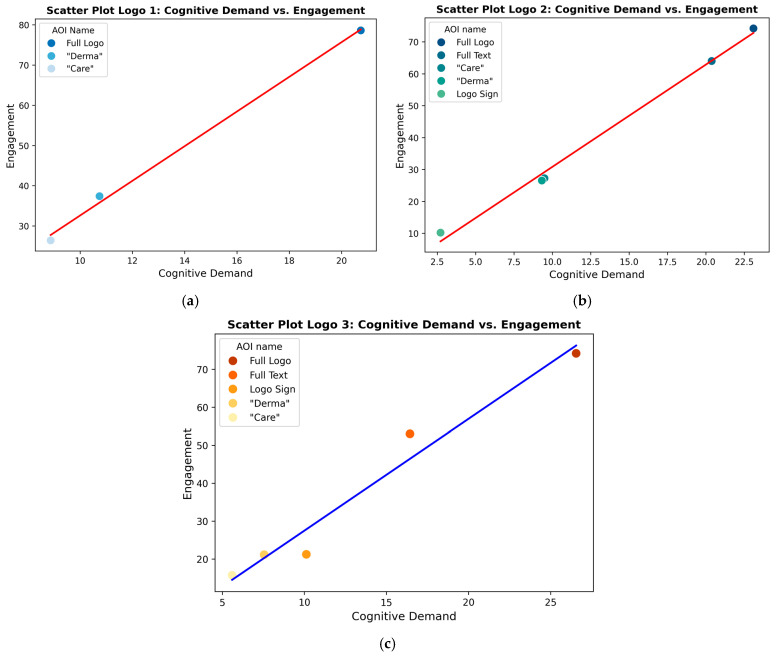
Comparative analysis of Total Attention across the three logos: scatter plots of Engagement vs. Cognitive Demand. (**a**) Logo 1: Relationship between Cognitive Demand and Engagement; (**b**) Logo 2: Scatter plot visualisation of Cognitive Demand versus Engagement; (**c**) Logo 3: Visualisation of Cognitive Demand versus Engagement. Each data point represents a specific Area of Interest (AOI), including elements such as “Full Logo”, “Full Text”, “Derma”, “Care”, and “Logo Sign”. Cognitive Demand scores (0–24) reflect the cognitive effort required to process the stimulus, while Engagement represents the intensity of user focus and motivational relevance. A lower Cognitive Demand indicates ease of processing, potentially resulting in shorter viewer attention. Higher Cognitive Demand (75–100) suggests more complex visual structure, which may sustain engagement. Linear regression lines (red and blue) are included to illustrate the statistical association between Cognitive Demand and Engagement. Colour differences are used solely for visual clarity across logo conditions and do not indicate methodological variance.

The study investigated the relationship between Cognitive Demand and Engagement to elucidate the influence of logo designs on viewer perception and cognitive processing (see Table 2 and Figure 3). Table 2 displays the Pearson Correlation coefficients between Cognitive Demand and Engagement for each logo design. The values for the Areas of Interest (AOIs) for each logo were correlated to examine the relationship between these two metrics. (The values for the Engagement and Cognitive Demand AOIs, along with their benchmark values, are presented in Table A1 and Table A2). A Pearson correlation test was employed to rigorously assess whether any variations in cognitive demand corresponded with changes in engagement, thereby providing insights into the concurrent movement of these two critical constructs. By quantifying the linear relationship, it is possible to determine if an increase in a logo’s cognitive demand consistently correlates with higher or lower levels of engagement. This methodology is particularly pertinent in the context of neuromarketing, where design elements simultaneously affect cognitive processing and emotional resonance. The resulting *p*-values further substantiate the reliability of the observed relationships, ensuring that the correlations are not attributable to chance and reinforcing their significance for informing logo design decisions. All correlations were robust and statistically significant (*p* < 0.01). Logo 1 demonstrated the highest correlation (r = 0.999), followed by Logo 2 (r = 0.997) and Logo 3 (r = 0.981). The Cognitive Demand metric in this study assesses the mental effort required by the logos. It identifies whether the logo simplifies understanding or introduces complexity, potentially forecasting clear communication or viewer confusion. Notably, Logo 1, which lacks dynamic elements, exhibits the highest correlation value (0.999, *p* = 9.959 × 10^−4^), suggesting that engagement and cognitive demand remain highly structured and predictable when the logo is static. However, this does not necessarily indicate higher emotional engagement, as static logos may induce a more neutral or stable response. In contrast, Logo 2, which incorporates dynamic elements in the word “Care”, exhibits a slightly lower correlation (0.997, *p* = 1.921 × 10^−4^). This subtle reduction suggests that minor dynamic elements introduce slight variability in how users engage with the logo, likely increasing cognitive demand without significantly altering the consistency of engagement. The most notable shift occurs in Logo 3, which features dynamic elements in the icon. It has the lowest correlation value (0.981, *p* = 2.925 × 10^−3^) among the three logos, indicating a more significant variation in user engagement. This result supports the hypothesis that dynamic elements, particularly in key visual features such as sub-icons, lead to increased cognitive demand and a less uniform engagement pattern, potentially reflecting higher emotional engagement and a more varied audience response (H2 partially supported). The findings partially corroborate Hypothesis 2, indicating that dynamic elements in logos elevate cognitive demand while sustaining high levels of engagement. The slightly diminished correlation observed for Logo 3 suggests that its dynamic icon may introduce more significant variability in engagement, potentially reflecting heightened emotional engagement. Although the differences in attention duration among the logos may appear minor, they can have substantial practical implications for brand recall:

1. Cumulative effect: Even modest increases in attention duration can accumulate over multiple exposures, potentially leading to enhanced brand recognition over time.
2. Quality of engagement: Logo 3’s more balanced attention distribution (2.9 s total, with 0.89 s on the icon) indicates a more comprehensive engagement with all the logo elements, which may contribute to improved overall brand recall.
3. Cognitive processing: The slight variations in attention duration may reflect differences in the depth of cognitive processing, with longer durations potentially indicating more thorough encoding of brand information.
4. Competitive advantage: In a crowded marketplace, even marginal improvements in attention retention could provide a competitive edge in brand recognition and recall. Research on cognitive engagement, such as the Rorschach task, suggests that complexity in visual stimuli is correlated with increased cognitive effort. Neurophysiological markers evidence this phenomenon, notably an increase in oxygenated haemoglobin in the prefrontal cortex, which is associated with higher cognitive load and emotional processing (Vitolo et al., 2021). Memory refers to the likelihood of an asset being retained in the viewer’s recollection after exposure based on visual distinctiveness and emotional resonance. Neuromarketing methodologies enable luxury brands to leverage consumers’ emotional and cognitive processes, thereby enhancing the efficacy of their marketing strategies. By understanding consumers’ emotional connections with a brand, marketers can develop campaigns that foster a stronger self-brand association. (J. Yu et al., 2023).

Logo 1, which lacks an icon, achieved a high full logo memory score (65.73%) that is entirely derived from textual recall, with a full-text score of 65.73%. Within the text, “Derma” (27.61%) and “Care” (25.45%) received nearly equal attention, suggesting that text-based logos facilitate a uniform distribution of memory. Nevertheless, the absence of a visual icon may constrain recall diversification, rendering the brand predominantly text-dependent. These measures enable luxury brands to evaluate the efficacy of their advertisements in terms of consumer recall and recognition, which is crucial for maintaining brand prestige (Baldo et al., 2022). In contrast, Logo 2, which incorporates a small “DC” sub-logo icon, exhibited a lower full logo memory score (51.9%) than Logo 1. The memory recall for the logo sign (7.5%) suggests that some attention was allocated to the sub-icon, resulting in reduced text recall (full text = 44.4%). Notably, both “Derma” (16.48%) and “Care” (19.9%) scored lower than in Logo 1, indicating that the addition of a minor logo sign may have fragmented the memory distribution, resulting in a weaker overall recall. Logo 3, which features a dynamic and prominent icon, achieved the highest full logo memory score (71.38%). Unlike Logo 2, its logo sign recall was significantly higher (23.17%), suggesting that the logo sub-icon played a dominant role in memory retention. However, this comes at the cost of a textual recall, as full-text memory decreased to 48.21%, with both “Derma” (17.35%) and “Care” (17.36%) receiving lower recall percentages than in Logo 1 (see Table 3). Table 3 presents a comparative analysis of the memory scores for the three logo designs, assessing recall performance across various Areas of Interest (AOIs), with H3 partially supported. The results partially corroborate Hypothesis 3, as Logo 3, characterised by its distinctive icon, demonstrated significantly superior outcomes in overall memory recall. Nevertheless, it is noteworthy that not all logos incorporating dynamic elements enhance customer recall equally. Understanding the relationship between memory retention and engagement is crucial in evaluating the effectiveness of logo designs. A robust correlation between these factors suggests that elements within a logo—whether static or dynamic—can impact its memorability and perceived engagement among viewers. These measures can differentiate between positive and negative consumer responses, providing luxury brands with valuable insights into the perception of their advertisements (Byrne et al., 2022). This section examines the Pearson Correlation values between memory and engagement scores for the three distinct logo designs, providing insights into how different design elements contribute to consumer perception and recall.

**Figure 4:**
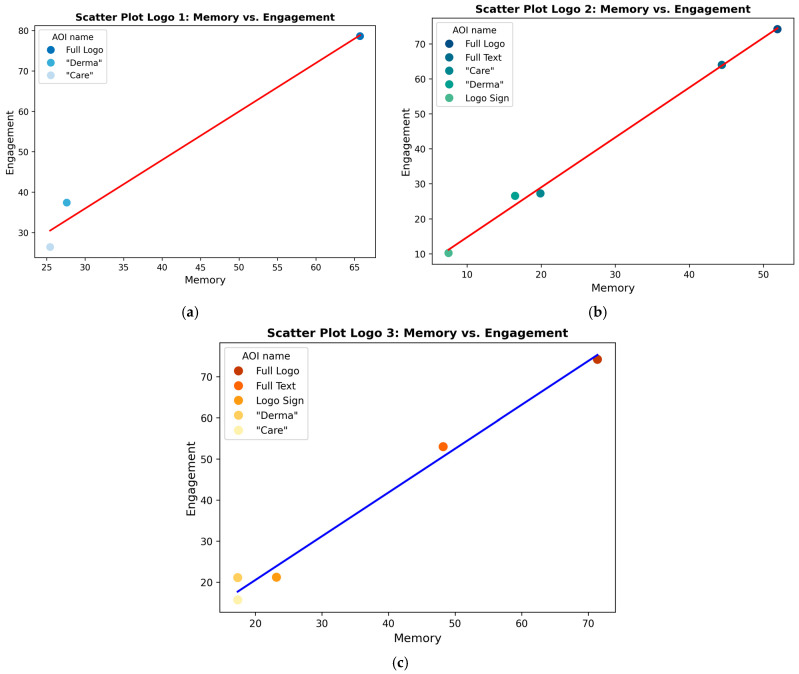
Comparative Analysis of Memory vs. Engagement across Three Logos: Scatter Plots. (**a**) Logo 1: Relationship between memory and engagement; (**b**) Logo 2: Scatter plot visualisation of memory versus engagement; (**c**) Logo 3: Visualisation of memory versus engagement. Data points reflect responses to individual AOIs, including “Full Logo”, “Full Text”, “Derma”, “Care”, and “Logo Sign”. The Memory metric captures the strength of encoding based on neurocognitive responses, while Engagement measures positive affective involvement such as interest, attention, and emotional resonance. Lower Memory scores (0–24) suggest minimal retention, medium scores (25-74) indicates moderate positive emotional response, whereas higher values (75–100) indicate deeper encoding and potential for long-term recall. Regression lines (red and blue) visually represent the linear correlation between memory and engagement. Colour variation between lines is employed solely to distinguish between logos for interpretive ease and carries no analytical significance.

Techniques that enhance memory retrieval, such as those involving norepinephrine neurons, can facilitate the recall of brand-related information. This is particularly pertinent for luxury brands, where emotional engagement and memory play a crucial role in consumer decision-making (Fukabori et al., 2020). The values of the Areas of Interest (AOIs) related to Memory and Engagement were correlated for each logo to examine the relationship between these two metrics. (For the AOI values for Engagement and Memory, along with their benchmark values, see Table A2 and Table A3). The Pearson Correlation was employed to investigate whether increased emotional involvement (engagement) corresponds to enhanced recall (memory) among viewers. This metric captures the linear relationship between these constructs, enabling the quantification of how variations in engagement influence long-term retention. This approach offers a comprehensive understanding of how design elements can foster both immediate interest and lasting impact. The Pearson Correlation between Memory and Engagement scores (see Table 4) reveals strong positive relationships across all three logo designs (see Figure 4). Logo 1 demonstrates a correlation value of 0.992 (*p* = 7.931 × 10^−3^), indicating that its static design facilitates a highly structured and predictable association between memory and engagement. Logo 2, with the highest correlation value of 0.998 (*p* = 8.5 × 10^−5^), suggests that the dynamic elements in its design—such as the motion effect in the word “Care”—enhance memory retention while maintaining engagement. Similarly, Logo 3 exhibits a correlation value of 0.994 (*p* = 5.29 × 10^−4^), although its scatter plot indicates slightly more variability, potentially due to a balance between its unique design features and viewer focus. The minimal difference in correlation among the three logos may indicate that the impact of dynamic elements on customer engagement and memory is limited, supporting H3 partially.

### 4.4. TensorFlow and Deep Learning Models in Saliency Mapping

Saliency map analysis is a robust technique for comprehending the distribution of visual attention across a logo design, elucidating which elements attract immediate focus and how gaze patterns evolve. Using colour transfer techniques predicated on salient feature mapping can enhance the visual appeal of logos by ensuring that colour schemes align with human visual preferences. This process involves creating saliency maps to identify attention-grabbing regions and adjusting colours accordingly (Wu & Xue, 2020). In this investigation, TensorFlow was utilised to generate saliency maps using deep learning models to predict regions of high visual importance through gradient-based backpropagation techniques. Specifically, Gradient-weighted Class Activation Mapping (Grad-CAM) was applied to visualise the most salient areas of each logo by computing the gradient of the predicted output with respect to the input image. This method highlights the areas that contribute most significantly to model predictions, providing a heatmap overlay that represents the intensity of viewer attention. Incorporating a confidence segmentation module in Class Activation Mapping (CAM) methodologies can facilitate the assessment of individual pixel contribution reliability, thus potentially improving the precision of object localisation (Kou et al., 2021). The saliency intensity values, represented by the colour bar ranging from 0.3 to 0.7, indicate the degree of attention allocated to different regions within the logo. The logos in this study were black against a white background, which influenced the saliency map distribution. Low saliency values (0.3–0.4, blue areas) are predominantly concentrated on the black elements of the logo, such as edges, shapes, or text, due to their contrast against the white background. Moderate saliency values (0.4–0.5, indicated by yellow/green areas) align with transitions between the logo and the background or secondary design elements. High saliency values (0.6–0.7, red areas) primarily represent the white background, which contributes minimal visual importance. This configuration emphasises the capacity of high-contrast elements in the logo to capture the viewer’s attention effectively. By analysing these saliency maps, one can assess the visual hierarchy, focal points, and cognitive demand imposed by each logo design. A well-structured saliency distribution suggests that a logo effectively directs attention to key brand elements, thereby enhancing recognition and recall. In contrast, a dispersed or unfocused distribution may indicate a need for design refinement. This investigation examines the saliency patterns of three distinct logo designs to assess how effectively they capture and direct viewer attention, offering insights into their potential influence on consumer perception and engagement. The subsequent analysis interprets the saliency results in detail, identifying strengths and areas for potential optimisation in each logo design.

To rigorously assess the degree of alignment in saliency patterns between logos, Pearson Correlation test was applied to the pixel intensities of their respective saliency maps. This method yields a singular statistic that indicates whether the distribution of attention intensities in one logo is linearly related to that of another. Essentially, higher correlation values suggest a more similar “attention footprint”, whereas lower correlations indicate distinct viewing patterns influenced by each logo’s unique visual characteristics. Table 5 presents the Pearson Correlation test results for pixel intensity comparisons between Logo 2 and Logo 1 and between Logo 3 and Logo 1, quantifying the similarity in attention distribution as depicted by their saliency maps. In the process of correlating pixel intensities between two 224 × 224 logo images, each comparison encompasses nearly 50,000 data points (224 × 224 = 50,176). This substantial sample size significantly enhances the statistical power of the analysis, resulting in extremely small *p*-values, even for modest correlations. Consequently, any non-zero correlation is likely to be detected, and the *p*-value may become so negligible that the software might display it as zero. Given the limited number of comparisons conducted, a formal multiple-comparisons correction (e.g., the Bonferroni or Benjamini–Hochberg method) was not applied. Even if such a correction were implemented, the *p*-values would remain exceedingly small, thereby not altering the overall conclusion of statistical significance. Therefore, the absence of a multiple-comparisons adjustment does not compromise the validity of the results, as the near-zero *p*-values are primarily attributable to the large sample size (tens of thousands of pixel-level data points) and the limited number of tests. The correlation value for Logo 2 vs. Logo 1 is 0.402 (*p* = 0.000 × 10^0^), indicating a statistically significant but relatively low correlation. This suggests that while certain areas may elicit similar attentional responses, the saliency patterns exhibit considerable divergence, potentially reflecting design differences. For Logo 3 vs. Logo 1, the correlation value is 0.155 (*p* = 1.536 × 10^−27^), which is statistically significant but markedly low, indicating a distinct attention distribution. The notable decrease in correlation in this instance suggests significant design variations between the two logo designs, supporting H1. The Pearson Correlation coefficient is a statistical measure that quantifies the strength and direction of the linear relationship between two variables, and its values range from −1 to +1. In this context, a coefficient of 1 signifies a perfect positive correlation, indicating identical attention patterns, while a coefficient of 0 denotes the absence of a linear correlation. Conversely, a coefficient of −1 represents a perfect negative correlation. The reported correlations (Logo 2 vs. Logo 1: 0.402; Logo 3 vs. Logo 1: 0.155) suggest weak to moderate positive correlations in attention patterns. The lower correlation between Logo 3 and Logo 1 implies a more distinct distribution of attention, likely due to the introduction of the dynamic icon. These coefficients quantify the degree of similarity or difference in viewer engagement with each logo design, providing insights into the impact of design variations on attention patterns.

**Figure 5:**
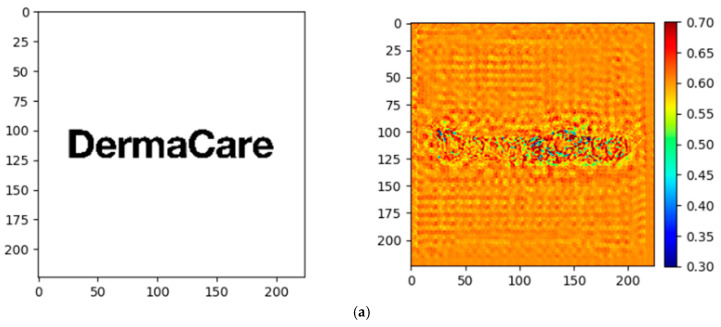

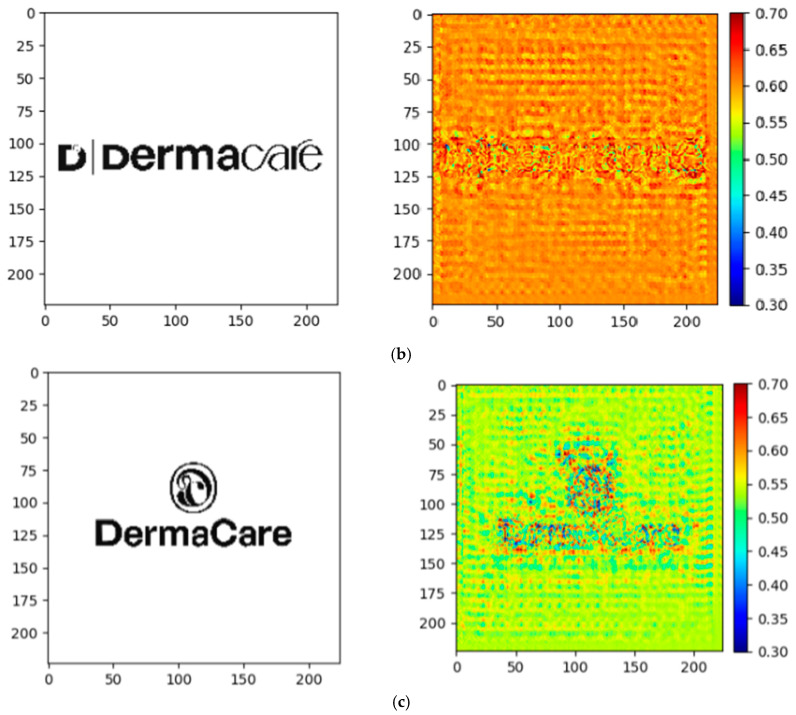
Comparative analysis of Memory vs. Engagement among the three logos: scatter plots. (**a**) Saliency map for Logo 1; (**b**) Saliency map for Logo 2; (**c**) Saliency map for Logo 3.

These findings elucidate the variability in saliency distribution across logos and underscore the influence of design elements, such as static vs. dynamic features, on attention patterns. The subsequent section will analyse the saliency map to identify the most prominent pixels of the logos. The saliency maps for the three logo designs (see Figure 5a–c) provide critical insights into the distribution of visual attention across different elements of each logo, elucidating their efficacy in capturing viewer engagement. Saliency is determined by calculating the gradient of the model’s output, specifically the predicted class score, concerning each pixel of the input image. Mathematically, this involves computing the partial derivative, which assesses how minor variations in each pixel’s intensity influence the model’s confidence in the selected class. Once these gradients are computed, they are normalised to values between 0 and 1 and subsequently represented as a heatmap. This gradient-based methodology is widely used to visualise the regions of an image that a deep neural network utilises when making its predictions. Logo 1 (see Figure 5a) exhibits a highly concentrated saliency pattern, with a significant amount of low saliency value (0.3–0.4) on the text “Care”, indicating that viewers’ gaze is immediately drawn to a dominant visual feature. This suggests that the logo establishes a clear focal point, enhancing brand recognition and memorability. In contrast, Logo 2 (see Figure 5b) displays a more diffused saliency pattern, with attention distributed across multiple areas rather than a single dominant focal point. This distribution suggests that while the logo contains several visually engaging elements, the absence of a clear primary focus may result in fragmented attention, potentially reducing immediate recognition and cognitive impact. Logo 3 (see Figure 5c) presents a more structured saliency distribution, with distinct high-attention areas that indicate a well-defined visual hierarchy. The low saliency pixels predominantly focus on the icon and “Derma” text. This suggests that the design effectively guides the viewer’s gaze in a sequential manner, facilitating a more intentional and controlled visual processing experience (H1 partially accepted). The interaction between endogenous (goal-directed) and exogenous (stimulus-driven) attention mechanisms can inform logo design strategies. Logos that align with consumer priorities (endogenous) while simultaneously eliciting spontaneous attention (exogenous) are likely to demonstrate increased efficacy (Jamoulle et al., 2022).

### 4.5. Emotional Survey Analysis

While emotions exert a substantial influence on decision-making processes, it is imperative to acknowledge the complexity and variability of emotional experiences (Holleman et al., 2021). To enhance the objectivity of the research, a survey was analysed to assess consumers’ emotional metrics. A spider chart in Figure 5 was constructed using 13 distinct emotions, which were measured and classified into four main categories. The following is a description of these emotions and the behavioural responses that these emotions may elicit. This classification provides a framework for interpreting the results.

Positive emotions (generally evoking or facilitating approach behaviour): Positive emotions generally lead to less impulsive decision-making (Manuel et al., 2020). They include the following: Happy: a general sense of well-being and contentment. Joyful: heightened happiness, often associated with pleasure and satisfaction. Excited: arousal and enthusiasm, stimulating anticipation and an active desire to engage.

Negative emotions (generally evoking avoidance behaviour): Negative emotions are associated with increased impulsivity due to reduced amygdala integrity (Manuel et al., 2020) and include the following:

Sad: a feeling of loss or disappointment, potentially reducing motivation to engage.

Worried: a state of concern or unease, typically triggering hesitation or risk aversion.

Confused: a lack of clarity or understanding, leading to cognitive overload or disengagement. Nervous: tension or uncertainty associated with fear of loss or making an incorrect decision.

Personal appeal (generally strengthening the positive emotional response): In the context of luxury branding, strategically employed aesthetic elements may be utilised to enhance the perceived value and exclusivity of a product, thereby eliciting a more emotionally engaging consumer experience (Bender et al., 2021). Personal appeal can be classified as follows: Seductive: a sense of allure and attraction that stimulates strong emotional engagement and desire. Luxury: a perception of exclusivity and prestige, evoking feelings of high status and aspiration. Special: a sense of uniqueness or personal significance, creating a feeling of being valued or chosen. Emotional brand attachment is a significant determinant of consumer behaviour towards luxury brands. It exerts more significant influence than brand involvement in fostering original brand patronage, as it engenders a more profound emotional connection with the brand (Bian & Haque, 2020).

General impact (generally strengthens all signals), which can include the following: Calm: a soothing and reassuring effect that reduces resistance and fosters a sense of comfort. Surprised: an unexpected element that captures attention, triggering curiosity and making the experience more memorable. The mitigation of emotional responses can potentially enhance affective attachment by fostering a positive and tranquil brand experience, which is deemed essential for luxury brands seeking to establish a robust emotional connection with consumers (Bian & Haque, 2020).

**Figure 6:**
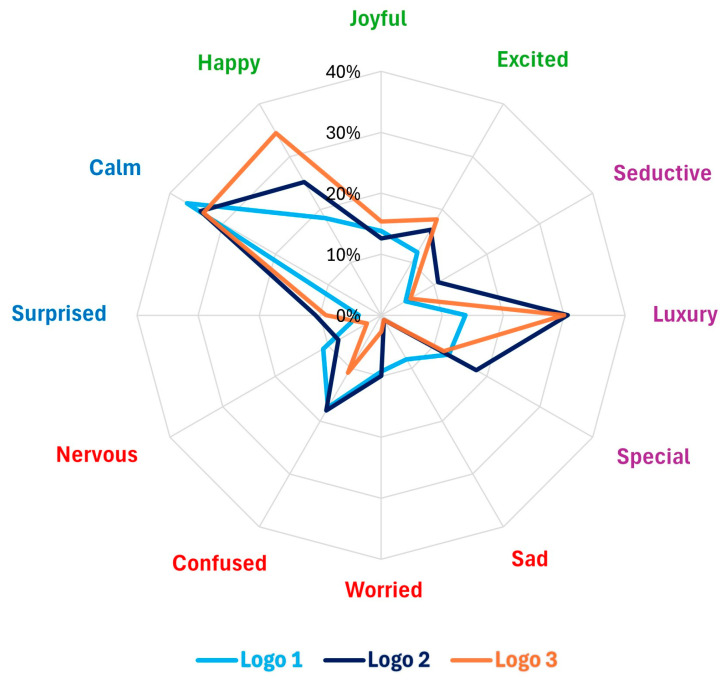
Comparative analysis of emotional responses to the three logo designs: metrics of Personal Appeal, Positive Emotions, and Negative Emotions. Radar chart depicting the emotional associations elicited by the three logo designs, based on participant ratings. Each axis represents a discrete emotional descriptor, with values indicating the proportion of participants who associated each emotion with a given logo. Emotions are grouped and color-coded by psychological valence and thematic category: Positive emotions (e.g., Joyful, Happy, Excited) are shown in green. Trust and calm-related emotions (e.g., Calm, Surprised) are shown in blue. Luxury and aesthetic emotions (e.g., Luxury, Special, Seductive) are shown in purple. Negative emotions (e.g., Sad, Worried, Confused, Nervous) are shown in red. This grouping aids interpretation by highlighting which logos are more frequently associated with positively or negatively valenced emotional responses, as well as aesthetic or trust-related impressions.

Logo 1 demonstrates inferior performance across all metrics except for Calm (36.7%). Notably, Logo 2 excels in three personal appeal emotions aligned with DermaCare’s business focus: Luxury (31%), Seductive (11%), and Special (18%), emphasising its strong ability to convey a premium and exclusive brand perception (see Figure 6).

Logo 2 also elicits the highest level of surprise from customers at 10.8%. Logo 3 performs moderately well across positive emotions such as Happy (35%), Joyful (15%), and Excited (18.2%), showcasing its uplifting emotional appeal (see Figure 6). This spider chart illustrates the emotional responses to the three logo designs across 13 distinct emotions. The chart is divided into four main categories: Positive Emotions, Negative Emotions, Personal Appeal, and General Impact. Key findings include Logo 2’s superior performance in personal appeal emotions (Luxury, Seductive, Special) and Logo 3’s strength in positive emotions (Happy, Joyful, Excited). All three logos demonstrate minimal impact (below 20%) regarding negative emotions. However, Logo 1 exhibits significantly higher scores in negative emotions such as Sad (8.3%) and Nervous (11%) compared with the others, further demonstrating its weaker overall emotional performance. Logo 3 elicits the least negative emotions across all metrics, which may be attributed to its uplifting appeal (see Figure 6). Dynamic logo elements significantly enhance emotional connections by adding vibrancy and sophistication to a brand’s visual identity. This perceived movement can elevate brand personality by suggesting activity and engagement, which are frequently associated with contemporary and innovative brands (Daniel, 2018). As evidenced by Logos 2 and 3, dynamic elements amplify positive emotions such as Luxury and Special and minimise negative responses, creating a more engaging and appealing brand experience. These dynamic features evoke a sense of motion and modernity, making the brand appear more innovative and relevant to consumers. In contrast, text-only designs such as Logo 1 fail to evoke the same level of emotional resonance, highlighting the critical role of dynamic elements in fostering stronger consumer–brand emotional bonds (H3 accepted). Dynamic imagery in logos increases consumer engagement, affecting attitudes towards the brand. Research on dynamic body–feature processing indicates that dynamic stimuli activate specific neural networks, suggesting that dynamic logos may similarly engage brain regions associated with visual and emotional processing (B. Li et al., 2022). This engagement is measured through self-reporting measures, demonstrating that perceived movement can enhance brand attitudes when congruent with brand characteristics. 

**Figure 7:**
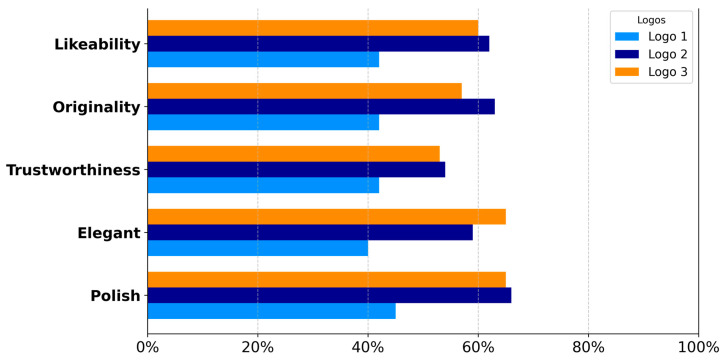
Comparative analysis of brand perception metrics for the three logo designs.

Our analysis demonstrates the performance of the three logos across five key metrics: Trustworthiness, Originality, Likeability, Elegance, and Polish. A horizontal bar chart (Figure 7) compares these brand perception metrics for the three logo designs. The chart clearly shows Logo 2’s overall strong performance across most metrics, while Logo 3 excelled in Elegance. Logo 1 underperformed across all the evaluated metrics, suggesting that it lacks appeal and credibility compared with the other designs. Logo 2, which incorporates a dynamic movement element in the text “Care”, emerged as the strongest overall performer, leading in Trustworthiness (66%), Originality (63%), Likeability (62%), and Polish (66%), indicating its comprehensive resonance with participants. Meanwhile, Logo 3 excelled in Elegance (65%), showcasing its refined design, mainly through the effective use of the icon positioned above the “DermaCare” text. Overall, incorporating dynamic elements into Logos 2 and 3 proved critical in enhancing the key attributes that closely aligned with DermaCare’s branding objectives (H3 accepted). Dynamic logo elements have a significant influence on industry association among participants, shaping perceptions, enhancing engagement, and impacting market performance. Dynamic logos can also enhance industry association by enabling organisations to swiftly adapt their visual identity to reflect industry trends and developments (Y. Li et al., 2022). The interplay between logo dynamism and product types demonstrates that dynamic logos are particularly effective for hedonic products, while static logos are more suitable for utilitarian products. Research indicates that dynamic and static visual stimuli can effectively convey category information in the brain (Robert et al., 2023). In advertising, static hedonic banners have been shown to elicit more robust neurocognitive processes, leading to enhanced recall. This phenomenon suggests that static logos may be more effective for specific product categories, particularly those that rely predominantly on emotional appeal (Casado-Aranda et al., 2022). This differentiation is crucial for aligning brand identity with consumer expectations. In this section, a stacked bar chart was utilised to analyse customer awareness of the brand and industry association. The survey provided options, including healthcare, cosmetics, luxury cosmetics, pharmaceuticals, and discounted goods.

**Figure 8:**
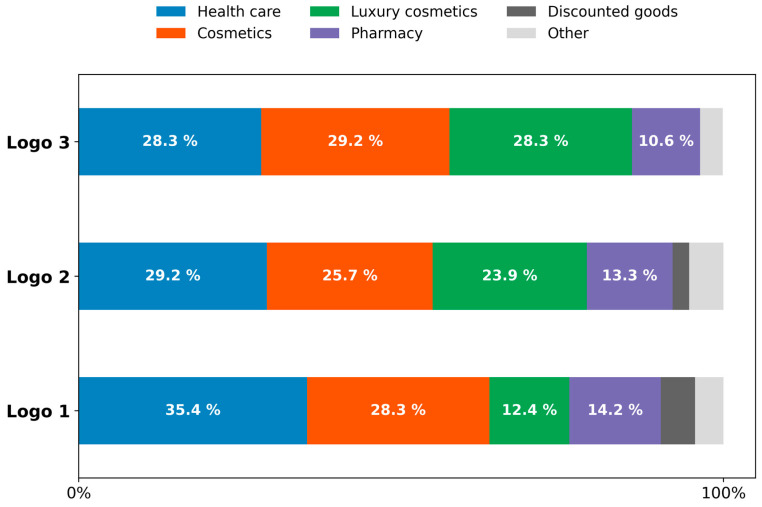
Industry association comparison.

Figure 8 illustrates the association of each logo with specific industries as perceived by participants. This stacked bar chart analyses customer awareness of the brand and the industry association for each logo. It presents associations with five industries: healthcare, cosmetics, luxury cosmetics, pharmaceuticals, and discounted goods. The chart effectively demonstrates how Logo 2 and Logo 3, with their dynamic elements, exhibit stronger associations with luxury cosmetics and the cosmetics industry compared with Logo 1. Logo 1 demonstrates the strongest association with healthcare (35.4%), which may be attributed to its simplicity and absence of dynamic elements, characteristics that are more closely aligned with traditional, stable industries. However, it exhibits lower associations with luxury cosmetics (12.4%) and cosmetics (28.3%). Conversely, Logo 2 and 3 display higher associations with luxury cosmetics, at 23.9% and 28.3%, respectively, indicating their appeal to premium and aesthetically oriented industries. Logo 3, featuring dynamic iconography, achieves a balanced association between luxury cosmetics (28.3%) and cosmetics (29.2%), suggesting its ability to resonate with high-end and mainstream markets. It also maintains relatively low associations with healthcare (28.3%) and pharmaceuticals (10.6%). Incorporating dynamic elements in logos significantly enhances industry associations by altering perception towards aesthetics-focused and high-end markets. As evidenced by Logo 3, the inclusion of dynamic iconography increases recognition in luxury cosmetics and cosmetics industries, establishing a stronger connection with premium branding. Dynamic iconography can influence brand stereotypes by enhancing a brand’s perceived competence and warmth. These stereotypes, in turn, affect consumers’ value perceptions and purchase intentions (Gidaković et al., 2022). In contrast, static designs, such as Logo 1, tend to align with more traditional industries, potentially limiting their versatility and contemporary appeal (H3 supported).

Traditional industries, such as finance, law, and manufacturing, often prioritise stability and reliability. Static logos, which remain unchanged over time, symbolise these values, providing reliability and credibility to clients and customers. (Song & Liao, 2023). The findings of this study provide substantial support for H4, demonstrating that a combined methodology of AI-powered eye tracking, EEG, and implicit and memory testing with qualitative cognitive surveys offers a more comprehensive assessment of logo performance. The eye-tracking and EEG data objectively capture subconscious attention patterns, fixation durations, and engagement levels, revealing how visual elements influence cognitive demand and memory retention. Eye-tracking data can also elucidate how attention is distributed among object features in working memory tasks, suggesting that attentional selection may enhance memory recall through the prioritisation of specific features (Printzlau et al., 2022), whilst EEG provides valuable insights into individual differences in memory retention by measuring neural responses during memory tasks (Sturm et al., 2023). Concurrently, the qualitative cognitive surveys provided explicit user feedback, offering insights into perceived engagement, emotional responses, and brand recall. Qualitative surveys rely heavily on interpreting open-ended responses, which can introduce bias and variability. In neuromarketing, EEG is often used to assess consumer preferences and brand associations. Research has demonstrated that electroencephalography (EEG) can elucidate insights not captured by conventional surveys, such as the N400 component’s capacity to detect brand-association strength (Camarrone & Van Hulle, 2019). By integrating both methodologies, the analysis identified discrepancies between implicit and explicit engagement. In contrast, the survey-based Engagement scores suggested moderate interest levels, and the Fast Response Time (FRT) data indicated significantly stronger emotional reactions, revealing the limitations of relying solely on self-reported measures. The Engagement score is derived using an advanced FRT (Fast Response Test) approach: Participants view the asset, whether an image or video, for a short duration (approximately 5 s). They are then given association words such as “Engaging”, “Interesting”, “Surprising”, “Attractive”, and “Happy” and must quickly respond with “yes” or “no” to these prompts. The score considers the yes/no answers, the speed of the responses (quicker responses suggest stronger associations), and the consensus among participants. For videos, the score is calculated for each frame and then combined. The final score is determined through sophisticated machine learning models trained on large datasets, linking visual features to human reactions. This technique offers a reliable measure of the asset’s capacity to elicit positive emotional engagement, focusing on immediate gut reactions rather than extended contemplation. This deep learning model is subsequently trained to predict scores based on comprehensive human-based research.

Additionally, the memory recall assessments demonstrated how text-based logos facilitate stronger word recall. In contrast, dynamic visual elements enhanced overall logo recognition, further validating the importance of utilising quantitative tracking and qualitative evaluation for a holistic understanding of logo effectiveness. Multi-response approaches, including perception-based and experience-based measures, have provided more profound insights into consumer preferences and product performance. These approaches can predict actual product choices more accurately than traditional methods that rely solely on hedonic responses (Lagerkvist et al., 2021). The results affirm the necessity of a multi-method approach for accurately assessing consumer responses to branding elements, thereby supporting the acceptance of H4. The findings provide support for hypotheses H1 to H4, with H1 being partially supported. Logos incorporating dynamic elements enhanced consumer attention in certain instances, though not consistently across all designs. Specifically, Logo 3, featuring a prominent dynamic icon, achieved the highest attention scores, whereas Logo 2, with subtle dynamic text elements, underperformed relative to the static Logo 1 in some metrics. 

H2 was partially supported. Dynamic elements in the logos increased cognitive demand while maintaining high engagement levels. However, the correlation between cognitive demand and engagement varied across logo designs, with Logo 3 exhibiting a slightly lower correlation, indicating more significant variability in engagement patterns. H3 was supported. Logos with dynamic elements, particularly Logo 3, demonstrated higher brand industry awareness and alignment with premium market segments. This was evidenced by superior memory scores, stronger associations with luxury cosmetics, and more positive emotional responses. H4 was also supported. The combined methodology of AI-powered eye tracking, EEG, implicit testing, and qualitative cognitive surveys provided a more comprehensive assessment of logo performance. This multi-method approach revealed insights that would not have been captured by a single methodology alone, validating the effectiveness of integrating neuroscientific and traditional research methods.

## 5. Discussion

### 5.1. Findings

The study revealed that logos with distinctive dynamic features, particularly those with visually prominent icons, consistently outperformed static or minimally dynamic designs across key metrics. Logo 3, featuring a dynamic and prominent icon, emerged as the most effective design, achieving the highest scores in total attention (103%), memory recall (71.38%), and positive emotional metrics, including happiness and luxury. Its structured saliency distribution facilitated balanced visual engagement, guiding viewers seamlessly between textual and iconographic elements. In contrast, Logo 1, while excelling in text-based memory recall (65.73%), lacked emotional resonance and engagement, underscoring the limitations of static designs. Logo 2, featuring dynamic text motion in the word “Care”, yielded moderate results, suggesting that minor dynamic elements alone are insufficient to drive a significant impact on consumers. The research found strong correlations between cognitive demand and engagement (see Table 2), as well as between memory and engagement (see Table 4), supporting the hypothesis that dynamic elements enhance consumer perception and interaction. However, not all dynamic logos equally improved brand performance (see Section 4.5). While Logo 3’s dynamic sub-icon effectively redistributed attention and amplified engagement, Logo 2’s subtle motion effects fell short of delivering comparable results. While survey results indicated moderate emotional engagement for Logo 2, the eye-tracking data revealed discrepancies in attention allocation, emphasising the value of combining these methodologies. These findings underscore the need for brands to strategically incorporate dynamic elements that align with their identity and target audience. By leveraging insights from saliency maps, emotional analysis, and attention metrics, businesses can optimise their logos to maximise engagement, foster emotional connections, and strengthen brand recall by employing high-end neuromarketing research models and sensors. The superior performance of Logo 3, which incorporated a dynamic and prominent icon, suggests that well-executed dynamic elements can significantly elevate a brand’s visual identity, leading to increased attention, stronger emotional resonance, and improved memory recall. By forecasting these preferences, luxury brands can tailor their offerings to align with the specific values of their target demographics (Wong & Park, 2023).

### 5.2. Implications

The practical applications of dynamic logo design for luxury premium brands encompass several critical aspects. The strategic incorporation of dynamic elements in logo design enhances consumer engagement, emotional connection, and brand recall. Brand managers in the luxury premium sector should prioritise visually distinctive and meaningful dynamic features to improve the effectiveness of brand identity. Regular assessment and refinement of logo performance using metrics such as attention distribution, emotional response, and memory recall are crucial for maintaining effectiveness in dynamic market environments. Saliency mapping can optimise the visual hierarchy of logo elements, ensuring that key brand components receive appropriate attention. Adapting logos for digital environments is crucial, as dynamic logo elements can significantly enhance an online brand’s presence and identity. Research indicates that incorporating emotional, conceptual, and situational appropriateness responses can enhance the prediction of consumption behaviour beyond traditional hedonic measures. (Giacalone et al., 2022). This research makes a significant contribution to the field of brand design and consumer behaviour, advancing it through its innovative approach and comprehensive findings. By integrating AI-powered eye tracking, EEG data, and traditional survey methods, the study establishes a new benchmark for logo evaluation methodologies. It quantifies the impact of dynamic logo elements on consumer attention, engagement, and brand perception, offering valuable insights for brand designers and marketers. By focusing on the luxury cosmetics sector, the study provides targeted insights for premium brands seeking to enhance their visual identity and market positioning. The study bridges neuroscience and marketing, demonstrating the value of integrating neuroscientific approaches with traditional marketing research to achieve a more comprehensive understanding of consumer responses to visual stimuli. The research offers practical implications for brand strategy, providing actionable insights for businesses to optimise their logo designs and potentially improve their brand recognition, emotional connection, and market performance. Furthermore, it contributes to the evolving theory of logo design in the digital age by examining the interplay between static and dynamic elements. The study’s methodological innovation, which combines AI eye tracking and EEG-based predictions of consumer behaviour with qualitative surveys, sets a new standard for research methodologies in brand perception studies. This multifaceted approach enhances the academic understanding of consumer–brand interactions and offers practical guidelines for businesses seeking to refine their visual identity.

### 5.3. Theoretical Contributions

The study makes significant theoretical contributions by establishing a new benchmark for logo evaluation methodologies through the integration of AI-powered eye-tracking, EEG data, and traditional survey methods. This approach bridges neuroscience and marketing, demonstrating the value of combining neuroscientific approaches with traditional marketing research for a more comprehensive understanding of consumer responses to visual stimuli. The research advances logo design theory in the digital age by examining the interplay between static and dynamic elements. The study’s methodological innovation establishes a new standard for research methodologies in brand perception studies, thereby enhancing the academic understanding of consumer–brand interactions. The integration of AI neuroscience high-end technology, employing prerecorded eye tracking, EEG, and implicit and memory testing on a large sample size (*n* = 300,000), along with qualitative surveys conducted on marketing selected segment groups, validates the effectiveness of the multi-method approach in capturing both explicit and implicit consumer responses. This research establishes a robust framework for evaluating logo performance by primarily using the neuromarketing model, setting a benchmark for future research and design innovations. The study’s comprehensive approach and innovative methodology make significant contributions to the fields of marketing, neuroscience, and brand perception.

### 5.4. Limitations

This study offers valuable insights into the impact of dynamic elements in logo design on consumer engagement and brand perception while acknowledging several limitations that necessitate further investigation. The research concentrated on a specific demographic of female participants aged 20–40 in the United States, which restricts its generalizability. Future research should incorporate a more diverse sample that includes various age groups, genders, and cultural backgrounds. The study’s focus on the luxury cosmetics sector also necessitates an expansion into other industries to achieve a more comprehensive understanding of the effects of dynamic logos across various market segments. The research primarily evaluated immediate responses to logo designs, underscoring the need for longitudinal studies to assess the long-term impact of dynamic logos on brand recall and consumer loyalty. Although advanced AI-powered eye tracking and EEG technologies were utilised, the predictive nature of these tools may not fully capture real-time human responses, indicating the potential benefit of incorporating live measurements in future research. The study’s limited scope regarding logo variations and contextual factors presents opportunities for more nuanced investigations. Future research should encompass a broader range of dynamic elements and logo variations and consider variables such as brand familiarity and market positioning. Additionally, expanding the analysis to examine how dynamic logos perform across various digital platforms and media types would offer valuable insights for practitioners in an increasingly multi-channel marketing discipline.

## 6. Conclusions and Recommendations

This study examined the influence of dynamic visual elements in logo design on consumer responses in the luxury brand marketing sector. By integrating advanced AI-based neuromarketing tools—including large-scale predictive eye tracking, EEG data, memory testing, and implicit measures—with qualitative emotional surveys, the study provided an interdisciplinary and multi-method analysis of logo effectiveness. The results demonstrated that dynamic logos, particularly those with distinct iconography, enhanced visual attention, cognitive engagement, and emotional resonance, thereby supporting their strategic importance in luxury branding contexts.

Among the three tested logo designs, Logo 3—which featured a visually prominent dynamic icon—consistently outperformed both the existing commercial logo (Logo 1) and the minimally dynamic typographic variant (Logo 2). These findings indicate that not all dynamic features produce uniform effects; the degree of salience and visual composition play a critical role in modulating consumer perception. Although Logo 2 included a dynamic element, its subtlety was insufficient to elicit comparable levels of viewer engagement or brand recall.

The following is an overview of the key research outcomes and hypotheses:

H1: Dynamic elements in logos enhance consumer attention: Partially supported. Logo 3 recorded the highest Start–End Attention (from 89.8% to 107%) and Total Attention (103.02%) scores, indicating a superior ability for capturing and sustaining focus. Logo 2, despite incorporating a dynamic typographic feature, underperformed, highlighting that attention gains are contingent on the salience and clarity of dynamic design elements.

H2: Logos with dynamic features increase cognitive demand and emotional engagement: Partially supported. Logo 3 exhibited increased cognitive demand alongside heightened emotional engagement scores, suggesting that distinct icons elevate the processing load while fostering emotional resonance. However, the correlation between cognitive demand and engagement in Logo 1 (r = 0.999) and Logo 2 (r = 0.997) remained higher than in Logo 3 (r = 0.981), indicating greater variability in engagement for more complex visual elements.

H3: Dynamic visual identity improves brand industry association and premium positioning: Supported. Memory scores and industry alignment ratings confirmed that dynamic icons (Logo 3) elicited stronger associations with luxury cosmetics and premium market segments, as reflected in increased top-two-box scores for emotions such as elegance, luxury, and feeling special.

H4: A hybrid methodology combining neuromarketing AI and qualitative survey methods provides a comprehensive assessment of logo performance: Supported. The integration of large-scale AI-predicted attention, engagement, and memory data with targeted qualitative survey insights yielded a multidimensional analysis, enhancing the interpretability and reliability of results. This combined framework proved effective in capturing both subconscious and reflective consumer responses.

To enhance the efficacy of luxury premium brand identities, it is advisable to incorporate visually distinctive and meaningful dynamic features within logo designs. These elements have been demonstrated to improve engagement, emotional connection, and brand recall. However, it is crucial to maintain a balance between dynamism and brand identity, ensuring that dynamic elements align with core values and target audience expectations while preserving a cohesive visual identity. Advanced testing methodologies, such as AI-powered eye tracking and EEG, as well as qualitative cognitive surveys, should be utilised to assess logo performance comprehensively. This multi-method approach captures both implicit and explicit consumer responses, addressing potential biases inherent in traditional single-method studies. The research has advanced previous methodologies by combining large-scale AI predictions (*n* = 300,000) with targeted qualitative surveys, thereby providing a more robust and representative analysis. Logos should be optimised for emotional impact, evoking positive emotions and aligning with the perceptions of luxury brands. This approach is supported by findings on the superior performance of certain logos in eliciting emotions such as luxury, seductiveness, and a sense of feeling special. To mitigate potential cultural biases, future studies should expand the demographic scope beyond the current focus on female US participants aged 20–40. Industry associations should be considered when designing logos, as the study demonstrated that well-designed dynamic features were more strongly associated with luxury cosmetics and premium markets. Investment in distinctive iconography is recommended to effectively redistribute attention and amplify engagement, as evidenced by specific logo performances in the study. Regular assessment and refinement of logo performance using metrics such as attention distribution, emotional response, and memory recall are essential to ensure ongoing effectiveness in dynamic market environments. Saliency mapping should be utilised to optimise the visual hierarchy of logo elements, ensuring that key brand components receive the appropriate attention. The study has improved upon traditional methods by incorporating AI-driven saliency analyses, providing more objective and detailed insights into visual attention patterns. Logos should be adapted for digital environments, as findings suggest that dynamic logo elements have the potential to enhance the online brand presence. Cognitive demand should be balanced by striving for logo designs that stimulate engagement without overwhelming consumers, as the study revealed a strong correlation between cognitive demand and positive engagement. To address potential biases in logo evaluation, future research should include the following: 1. Expand the sample to include diverse age groups, genders, and cultural backgrounds. 2. Conduct longitudinal studies to assess the long-term impacts of dynamic logos on brand recall and consumer loyalty. 3. Incorporate live measurements in addition to AI predictions to capture human responses more accurately in real time. 4. Examine a broader range of dynamic elements and logo variations to account for design nuances. 5. Investigate how dynamic logos perform across various digital platforms and media types to provide more comprehensive insights for multi-channel marketing strategies.

### 6.1. Strategic Recommendations

Luxury brands should implement visually salient dynamic icons to optimize attentional capture and emotional engagement. Logo complexity requires careful calibration to balance cognitive load and engagement benefits. Evaluation frameworks should integrate neuromarketing metrics with qualitative perception analyses to assess both conscious and subconscious responses. Demographic specificity in neuromarketing evaluations is crucial for developing market-specific visual strategies that enhance brand resonance and alignment.

### 6.2. Future Research Directions

Investigate the longitudinal effects of dynamic logos on brand loyalty and repeated exposure. Expand testing beyond the US female demographic and apply neuromarketing protocols in dynamic environments, such as video-based media and in-store digital displays, to improve generalizability. Enhance the adaptability of AI neurometrics across linguistic and cultural contexts to advance the field of neuromarketing.

This study contributes to the evolving field of consumer neuroscience by illustrating how neuromarketing methodologies, when properly integrated, can decode the cognitive and emotional mechanisms behind visual branding. The methodological innovations and empirical findings outlined here offer valuable tools for both academic researchers and practitioners aiming to design and evaluate logos that resonate in high-value, attention-competitive markets. By implementing these recommendations and addressing potential biases, luxury premium brand managers can create more impactful and memorable brand identities that resonate with their target audience, ultimately strengthening brand equity and market performance.

## Figures and Tables

**Table 1:** Comparative analysis of time allocation across three logo designs.

			AOI’s Identification		
Logo Design	Full Logo	Logo Sign	Full Text	“Derma”	“Care”
1	2.29	-	2.29	1.41	0.88
2	2.51	0.18	2.33	1.41	0.6
3	2.9	0.89	2.01	0.93	0.49

**Table 2:** Pearson Correlation test results for Engagement and Cognitive Demand scores across the three logo designs.

Logo Design	Correlation Value	*p*-Value	Statistical Significance
Logo 1	0.999	9.959 × 10^−4^	Significant
Logo 2	0.997	1.921 × 10^−4^	Significant
Logo 3	0.981	2.925 × 10^−3^	Significant

**Table 3:** Memory score comparison among the three logos.

Logo Design	AOI Names				
	Full Logo	Logo Sign	Full Text	“Derma”	“Care”
1	65.73	-	65.73	27.61	25.45
2	51.9	7.5	44.4	16.48	19.9
3	71.38	23.17	48.21	17.35	17.36

This table compares the memory scores for the three logo designs, evaluating recall performance across different Areas of Interest (AOIs). The Full Logo score indicates overall brand retention, while scores for subcomponents such as Logo Sign and Full Text highlight specific areas of attention and recall. The Memory score indicates the likelihood that viewers will recall the asset after seeing it based on its visual uniqueness and emotional impact. A high Memory score suggests that customers are more likely to remember the asset for an extended time. Memory Scores are measured on a scale from 0 to 100: Low scores (0–40) indicate that the asset is not very memorable, and viewers are unlikely to remember it after seeing it. Medium scores (41–70) indicate that the asset has average memorability, with some viewers likely to recall it. High scores (71–100) indicate that the asset is highly memorable, and most viewers are likely to recall it after exposure.

**Table 4:** Pearson Correlation Coefficients Between Engagement and Memory Scores for Three Logo Designs.

Logo Design	Correlation Value	*p*-Value	Statistical Significance
Logo 1	0.992	7.931 × 10^−3^	Significant
Logo 2	0.998	8.5 × 10^−5^	Significant
Logo 3	0.994	5.29 × 10^−4^	Significant

**Table 5:** Pearson Correlation coefficients for pixel intensity comparisons for Logo 2 vs. Logo 1 and Logo 3 vs. Logo 1.

Logo Comparison	Correlation Value	*p*-Value	Statistical Significance
Logo 2 vs. Logo 1	0.402	0.000 × 10^0^	Significant
Logo 3 vs. Logo 1	0.155	1.536 × 10^−27^	Significant

## Data Availability

The original contributions presented in this study are included in the article. For further inquiries, please contact the corresponding author.

## Abbreviations

The following abbreviations are used in this manuscript:

OBC	Oxford Business College
AI Eye Tracking	Predict
AI EEG	Predict
AOI	Area of Interest
FRT	Fast Response Test

## Appendix A

**Table A1:** Table of AOI scores regarding engagement for each logo.

Logo Design	AOI Identification–Engagement Scores				
	Full Logo	Logo Sign	Full Text	“Derma”	“Care”
1	78.61	-	37.39	26.39	6.05
2	74.2	10.21	63.99	26.54	27.25
3	74.21	21.21	53	21.12	15.69

(Insignificant value: 0–53.4; Significant value: 54.5–100).

**Table A2:** Table of AOI scores regarding cognitive demand for each logo.

Logo Design	AOI Identification–Cognitive Demand Scores				
	Full Logo	Logo Sign	Full Text	“Derma”	“Care”
1	20.74	-	10.74	8.87	20.74
2	23.1	2.72	20.38	9.32	9.49
3	26.55	10.12	16.43	7.55	5.6

(Significant value: 0–31.3; Insignificant value: 31.4–100).

**Table A3:** Table of AOI scores regarding memory for each logo.

Logo Design	AOI Identification–Memory Scores				
	Full Logo	Logo Sign	Full Text	“Derma”	“Care”
1	65.73	-	27.61	25.45	65.73
2	51.88	7.5	44.38	16.48	19.9
3	71.38	23.17	48.21	17.35	17.36

(Insignificant value: 0–68.4; Significant value: 68.5–100).
